# 18β-glycyrrhetinic acid inhibits lung adenocarcinoma progression by targeting the MET/AKT signaling axis

**DOI:** 10.3389/fcell.2026.1752632

**Published:** 2026-08-21

**Authors:** Gongyu Wu, Huilin Guan, Mengyao Lv, Zhiqiang Chen, Changjiu Gao, Wanzhen Su, Hui Fu

**Affiliations:** School of Pharmacy, Mudanjiang Medical University, Mudanjiang, China

**Keywords:** 18β-glycyrrhetinic acid, bioinformatics, lung adenocarcinoma, MET, PI3K/AKT signaling pathway

## Abstract

**Background:**

18β-Glycyrrhetinic acid (18β-GA), a major bioactive triterpenoid from licorice, has demonstrated anti-cancer potential; however, its mechanisms against lung adenocarcinoma (LUAD) remain unclear.

**Methods:**

This study employed an integrated approach combining network pharmacology, bioinformatics, and experimental validation to investigate the anti-LUAD efficacy and underlying molecular targets of 18β-GA.

**Results:**

*In vitro* and *in vivo* assays demonstrated that 18β-GA dose-dependently inhibited LUAD cell proliferation, migration, and colony formation, while significantly suppressing tumor growth in a Lewis lung carcinoma (LLC) allograft model. By integrating network pharmacology with TCGA transcriptomic data, MET was identified as a core upstream target, and the PI3K/AKT signaling pathway was revealed as a significantly enriched pathway. Molecular docking and molecular dynamics simulations revealed that 18β-GA forms stable, high-affinity complexes in the catalytic pockets of both MET and AKT. Subsequent biological validations confirmed that 18β-GA attenuated the phosphorylation of critical signaling components, including MET, PI3K, AKT, and mTOR, without altering their mRNA or total protein expression. Crucially, functional rescue assays revealed that exogenous MET activation via its specific ligand HGF simultaneously reversed the 18β-GA-induced suppression of both p-MET and p-AKT. In contrast, the AKT agonist SC79 effectively restored p-AKT levels and reversed the anti-tumor efficacy of 18β-GA, but it failed to rescue the inhibition of upstream p-MET. This unidirectional reversal rigorously validates MET as the upstream regulator of AKT.

**Conclusion:**

18β-GA suppresses LUAD by targeting upstream MET to drive a top-down cascade blockade of the PI3K/AKT/mTOR signaling pathway, providing a pharmacological rationale for its application in LUAD targeted therapy.

## 1 Introduction

LUAD is the predominant histological subtype of lung cancer, accounting for approximately 40% of cases globally (Nagy-Mignotte et al., 2011; Lee et al., 2014). Lung cancer remains the leading cause of cancer-related mortality worldwide (GLOBOCAN, 2020), accounting for approximately 1.76 million deaths annually (Sung et al., 2021). The incidence of LUAD has increased significantly, particularly in developing countries, due to increased exposure to environmental carcinogens (Chaitanya Thandra et al., 2021). Current treatments, including platinum-based chemotherapy, EGFR tyrosine kinase inhibitors, and PD-1/PD-L1 immune checkpoint inhibitors, improve patient outcomes but are hampered by a limited duration of response and frequent toxicities (Xia et al., 2017; Shen et al., 2018; Zhang et al., 2023). These toxicities are common, with approximately 60%–70% of patients experiencing grade ≥3 adverse events such as chemotherapy-induced myelosuppression, radiation pneumonitis, and immune-related endocrinopathies, which limits long-term treatment use (Li et al., 2011; Lee et al., 2023).

The PI3K/AKT signaling pathway is central to lung cancer initiation and progression, making it a crucial target for therapeutic interventions (Sarris et al., 2012). Dysregulated activation of this pathway is significantly associated with a higher clinical stage, poor differentiation, and lymph node metastasis, and notably, significantly shorter overall survival in LUAD patients, highlighting its value as a prognostic biomarker (Jiang et al., 2014; De Marco et al., 2017; Fan et al., 2024). Beyond PI3K/AKT, aberrant activation of the MET receptor tyrosine kinase is another key therapeutic target in lung cancer. Upon binding to its ligand hepatocyte growth factor (HGF), MET activates multiple downstream pathways, including the PI3K/AKT pathway, to regulate cell proliferation, migration, invasion, and angiogenesis, thereby driving tumorigenesis and progression (Organ and Tsao, 2011; Mo and Liu, 2017; Comoglio et al., 2018). Consequently, targeting MET not only directly inhibits its kinase activity but also blocks this critical downstream pathway, offering a more comprehensive approach to suppress tumor growth and survival (Scagliotti et al., 2013). Therefore, developing agents that co-target both MET and PI3K/AKT presents a promising strategy for treating cancers driven by this interconnected signaling network.

Botanical drugs play a crucial role in the development of natural anti-cancer therapies (Efferth et al., 2007). Many studies have shown that plant-derived bioactive compounds can selectively inhibit malignant tumor cell proliferation while sparing normal tissues (Aggarwal et al., 2007). 18β-GA, the primary triterpenoid in licorice, exhibits a wide range of pharmacological effects (Jiang et al., 2026). 18β-GA confers anti-inflammatory effects by inhibiting the NF-κB pathway and demonstrates antioxidant activity by activating the Nrf2 pathway (Cai et al., 2018; Chen et al., 2024; Wang et al., 2024). Furthermore, it exerts broad-spectrum antitumor effects by inducing endoplasmic reticulum stress, promoting mitochondrial apoptosis via Bcl-2/Bax modulation, and polarizing tumor-associated macrophages toward the M1 phenotype (Wang et al., 2016; Jin et al., 2023). Its efficacy has been demonstrated in various tumor models: inducing apoptosis in MCF-7 breast cancer cells, downregulating cyclin D1 in hepatocellular carcinoma, and inhibiting the Wnt/β-catenin pathway in colorectal cancer (Hasan et al., 2016; Wang et al., 2017; Hsu et al., 2023). In LUAD, 18β-GA induces apoptosis by modulating related proteins—increasing pro-apoptotic Bax and Bim, decreasing anti-apoptotic Bcl-2, and activating caspases-9 and -3. It also triggers G_2_/M cell cycle arrest via p21 and p27 upregulation, thereby suppressing proliferation, and inhibits the MAPK pathway by reducing ROS levels (Huang et al., 2014; Zhu et al., 2015; Luo et al., 2021). Despite these findings, the interactions between 18β-GA and key oncogenic drivers, particularly MET and the PI3K/AKT signaling pathway, remains poorly understood. Systematic investigation is essential to elucidate the regulatory mechanisms and functional impact of 18β-GA on this critical signaling axis.

Traditional network pharmacology is limited by its inability to capture tissue-specific gene expression, correlate with clinical prognosis, or resolve complex signaling pathway crosstalk. To overcome these limitations, we adopted an integrative multi-omics approach that combines clinically guided bioinformatic analysis of the TCGA-LUAD transcriptome with rigorous *in vitro* and *in vivo* validation. This strategy improves the accuracy of 18β-GA target identification and directly links pathway modulation to clinical outcomes. Elucidating 18β-GA’s action on the MET/AKT axis enables new therapeutic strategies for MET-driven and PI3K/AKT-pathway activated LUAD. By integrating botanical medicine with molecular oncology, this study provides a robust theoretical and experimental foundation for developing 18β-GA as a targeted therapy for LUAD.

## 2 Materials and methods

### 2.1 Cell lines and cell culture

The following cell lines were used: human LUAD A549 (CL-0016, Procell, China), NCL-H1299 (H1299, C6348, BDBIO, China), murine Lewis lung carcinoma (LLC, CL-0140, Procell, China), and human bronchial epithelial BEAS-2B (C5382, BDBIO, China). A549 and H1299 cells were cultured in RPMI-1640 medium (C22400500BT, Gibco, United States) supplemented with 10% heat-inactivated fetal bovine serum (FBS, 164,210, Procell, China). LLC and BEAS-2B cells were maintained in DMEM (C11995500BT, Gibco, United States) with 10% FBS.

### 2.2 Cell viability assays

18β-GA (HY-N0180, MedChemExpress, United States) was dissolved in DMSO. Cells were seeded, allowed to adhere, and treated with 18β-GA (0, 20, 40, 60, 80, and 100 μM) for 24 h. Cell viability was assessed using the Cell Counting Kit-8 (CCK-8; CK04, Dojindo, Japan) per the manufacturer’s protocol. The IC_50_ value for A549 cells was determined, based on which a high (IC_50_) and a low (0.5 × IC_50_) dose were selected for subsequent experiments.

### 2.3 Cell migration assays

Cell migration was assessed using Transwell chambers. Briefly, 7.0 × 10^4^ serum-starved cells were seeded in the upper chamber with serum-free medium, while the lower chamber contained medium with 10% FBS as a chemoattractant. Both chambers were treated with high or low doses of 18β-GA. After 24 h, non-migrated cells were removed; migrated cells were fixed, stained with 0.1% crystal viole. The stained cells were then imaged and counted using a light microscope (DM1000, Leica, Germany).

### 2.4 Cell clone formation assays

Cells were trypsinized, counted, and seeded at 1 × 10^3^ cells per well in 6-well plates. After 7 days of culture with medium refreshed every 2–3 days, colonies were fixed with 4% paraformaldehyde, stained with 0.1% crystal violet and photographed.

### 2.5 Immunofluorescence staining

Cell proliferation activity was evaluated by Ki67 immunofluorescence staining. Cells seeded on coverslips were treated with 18β-GA at low and high concentrations for 24 h. After treatment, cells were fixed with 4% paraformaldehyde (G1101, Servicebio, China) for 15 min at room temperature, permeabilized with 0.5% Triton X-100 (GC204003, Servicebio, China) for 10 min, and blocked with 5% bovine serum albumin (BSA, GC305010, Servicebio, China) for 1 h. Samples were incubated with anti-Ki67 primary antibody (27309-1-AP, Proteintech, China) at 4 °C overnight, followed by incubation with fluorescent secondary antibody (RGAR002, Proteintech, China) for 1 h at room temperature protected from light. The coverslips were mounted with an antifade mounting medium containing DAPI (S2110, Solarbio, China), and images were acquired using a fluorescence microscope.

### 2.6 Establishment of the lewis lung cancer model and drug administration

Twenty male C57BL/6 mice (4–6 weeks old, 16–18 g) were purchased from Charles River (China) and housed under specific pathogen-free (SPF) conditions. An LLC model was established by subcutaneous inoculation of each mouse with 1 × 10^7^ LLC cells (in 0.2 mL of PBS) into the right flank (Figure 2A). Tumor size was measured every 48 h using digital calipers, and volume was calculated using the formula (length × width^2^)/2. On day 5 post-inoculation, when palpable tumors had formed, the mice were randomly divided into five groups (n = 4) using a random number table (Figure 2B): Model: saline (0.2 mL, ip, daily); 18β-GA low-dose (LD): 18β-GA (30 mg/kg, ip, daily); 18β-GA high-dose (HD): 18β-GA (60 mg/kg, ip, daily); DDP: cisplatin (3 mg/kg, ip, every 3 days); Combination: 18β-GA (60 mg/kg, ip, daily) + cisplatin (3 mg/kg, ip, every 3 days). The treatment period lasted for 14 days. Subsequently, all mice were deeply anesthetized by ip injection of pentobarbital sodium (50 mg/kg) and euthanized. Tumor tissues were excised, weighed, photographed, and processed for further analysis.

### 2.7 Network pharmacology and bioinformatics

#### 2.7.1 Acquisition of drug and disease targets

Potential targets 18β-GA were retrieved from four databases: Traditional Chinese Medicine Systems Pharmacology (TCMSP; https://www.tcmsp-e.com/tcmsp.php), ETCM2.0 (http://www.tcmip.cn/ETCM2/front/#/), SwissTargetPrediction (http://www.swisstargetprediction.ch/), and PharmMapper (https://www.lilab-ecust.cn/pharmmapper/). Gene nomenclature was subsequently standardized using the UniProt database (https://www.uniprot.org/).

Disease targets associated with LUAD were acquired from three databases: GeneCards (https://www.genecards.org/), OMIM (https://omim.org/), and DisGeNET (https://disgenet.com/).

#### 2.7.2 Identification of overlapping targets and functional enrichment analysis

The putative targets of 18β-GA and LUAD-associated targets were imported into the Xiantao Academic platform (www.xiantao.love) to identify common targets and construct a Venn diagram. Subsequently, these overlapping targets were subjected to Gene Ontology (GO) and Kyoto Encyclopedia of Genes and Genomes (KEGG) pathway enrichment analyses using the DAVID tool (https://david.ncifcrf.gov) and the REACTOME database (https://reactome.org). *Homo sapiens* was set as the background species, with a significance threshold of *p* < 0.05. The enrichment results were plotted by https://www.bioinformatics.com.cn, an online platform for data analysis and visualization.

#### 2.7.3 PPI network construction and hub genes identification

Overlapping genes between 18β-GA and LUAD targets were imported into the STRING database (https://cn.string-db.org/) to construct a protein-protein interaction (PPI) network using a high confidence interaction score (0.900). The network was visualized using Cytoscape (v3.10.1) software, with nodes ranked according to Degree centrality. Hub genes were further analyzed and screened using the CytoHubba plugin within Cytoscape (v3.10.1). To enhance result reliability, 11 topological analysis methods were comprehensively applied: Betweenness, Bottleneck, Closeness, Degree, DMNC, Eccentricity, EPC, MCC, MNC, Radiality, and Stress.

#### 2.7.4 Analysis of TCGA-LUAD transcriptomic data

Transcriptomic and clinical data for LUAD were retrieved from the TCGA database (https://www.cancer.gov/ccg/research/genome-sequencing/tcga). This dataset includes tumor and normal tissue from LUAD patients. Differential expression analysis was performed using the DESeq2 package in R (v4.3.2) to identify differentially expressed genes (DEGs). Genes with an adjusted *p*-value <0.05 and absolute log_2_ fold change >1 were considered statistically significant.

#### 2.7.5 Data screening and analysis of key genes

The overlapping genes between DEGs and hub genes were identified and visualized in a Venn diagram using the Xiantao Academic platform (https://www.xiantao.love/). The expression of these overlapping genes was then validated at the transcriptomic level using the GEPIA2 database (http://gepia2.cancer-pku.cn/#index), which integrates data from TCGA and the GTEx project to improve reliability. Genes with a statistically significant differential expression (*p* < 0.05) were subjected to overall survival (OS) analysis using the Kaplan-Meier Plotter platform (https://kmplot.com/analysis/). Genes demonstrating a significant correlation with poor prognosis (logrank *p* < 0.05) were defined as key genes. Finally, to further corroborate the differential expression of these key genes, a paired analysis was conducted by comparing tumor tissues with normal tissues from the TCGA-LUAD cohort using the “limma” and “ggpubr” R packages (v4.3.2).

#### 2.7.6 Clinical Correlation analysis of key genes

Patients from the TCGA-LUAD cohort were stratified into high and low key gene expression groups based on the median expression value. This stratification was visualized using the “ComplexHeatmap” package in R (v4.3.2). The associations between these expression groups and clinicopathological characteristics (e.g., age, sex, overall stage, and TNM stage) were then assessed using the Chi-square test.

#### 2.7.7 Molecular docking

The two-dimensional (2D) structure of 18β-GA was retrieved from the PubChem database (http://pubchem.ncbi.nlm.nih.gov/) and converted into its three-dimensional (3D) conformation using ChemOffice software. The resulting 3D structure was energy-minimized and saved in MOL2 format for docking studies. High-resolution crystal structures of target proteins were downloaded from the RCSB Protein Data Bank (PDB; http://www.rcsb.org/) to serve as receptor templates. These structures were preprocessed using PyMOL (v2.5) to remove water molecules, ions, and any co-crystallized ligands, retaining only the protein chains of interest. The cleaned structures were then saved in PDB format. These structures were preprocessed using PyMOL (v2.5) to remove water molecules, ions, and any co-crystallized ligands, retaining only the protein chains of interest. The cleaned structures were then saved in PDB format.

#### 2.7.8 Molecular dynamics simulation

Molecular dynamics (MD) simulations were performed using the GROMACS 2022 software package. Topologies and force field parameters for the protein receptor were generated using the pdb2gmx tool and assigned according to the CHARMM36 force field. Parameters for the ligand were obtained from the CGenFF program. The receptor protein was parameterized with the CHARMM36 force field, while the ligand was described using CGenFF (Jo et al., 2008). The solvated system was built by embedding the protein-ligand complex in a cubic TIP3P water box, with a minimum distance of 1.0 nm between the solute and the box boundary. The system was neutralized by adding appropriate ions using the gmx genion tool (Mark and Nilsson, 2001). Long-range electrostatic interactions were treated using the Particle Mesh Ewald (PME) method with a Fourier spacing of 0.12 nm and a direct space cutoff of 1.0 nm. Covalent bonds involving hydrogen atoms were constrained using the LINCS algorithm. Simulations were integrated using the Verlet leapfrog algorithm with a time step of 2 fs. Energy minimization was conducted in two stages. First, the system was minimized for 3,000 steps using the steepest descent algorithm with positional restraints applied in a stepwise manner: initially, only water molecules were energy-minimized while restraining the solute atoms, followed by minimization of the entire system while restraining counterions. Second, an additional 2,000 steps of conjugate gradient minimization were performed without any restraints. Following minimization, production molecular dynamics simulations were carried out for 100 ns in the NPT ensemble at 310 K. The resulting trajectories were analyzed to evaluate the system’s stability and energetics. The following properties were calculated using the specified GROMACS tools: Root-mean-square deviation (RMSD) using gmx_rmsd; Root-mean-square fluctuation (RMSF) using gmx_rmsf; Hydrogen bonds (H-bonds) using gmx_hbond; Radius of gyration (Rg) using gmx_gyrate; Solvent-accessible surface area (SASA) using gmx_sasa; Free energy landscape (FEL) using gmx_sham.

### 2.8 Histological analysis of tumor tissue

Tumor tissue samples were fixed in 4% paraformaldehyde for 48 h at room temperature, dehydrated through a graded ethanol series, cleared with eco-friendly deparaffinization solution (G1128, Servicebio, China), and embedded in paraffin. Serial sections of 5 μm thickness were prepared, followed by deparaffinization and rehydration. The sections were stained with Hematoxylin and Eosin (H&E, G1005, Servicebio, China) according to standard protocols, followed by dehydration, clearing, and mounting with a synthetic resinous medium. Subsequently, the slides were imaged using a light microscope (DM1000, Leica, Germany).

### 2.9 Tissue immunohistochemistry (IHC)

Tumor sections (5 μm thickness) were deparaffinized using an eco-friendly clearing agent and rehydrated through a graded ethanol series. Antigen retrieval was performed in citrate buffer. Endogenous peroxidase activity was blocked by incubating sections with 3% H_2_O_2_ for 10 min. Sections were then incubated with a primary anti-Ki67 antibody overnight at 4 °C, followed by incubation with an HRP-conjugated secondary antibody. Color development was carried out using a DAB chromogen kit (E-IR-R101, Elabscience, China). Finally, sections were counterstained with hematoxylin, dehydrated, mounted, and imaged using a light microscope (DM1000, Leica, Germany).

### 2.10 Reverse transcription quantitative real-time polymerase chain reaction (RT-qPCR)

Total RNA was isolated with the Total RNA Isolation Kit (RC112, Vazyme, China). The concentration and purity of RNA were measured using a NanoDrop spectrophotometer. cDNA was synthesized from 1 μg of RNA with RT SuperMix containing gDNA Remover (R202, EnzyArtisan, China) under the following conditions: 37 °C for 15 min, and 85 °C for 5 s for enzyme inactivation. Quantitative PCR was performed using ChamQ Universal SYBR Master Mix (Q711, Vazyme, China). Each 20 μL reaction mixture consisted of 10 μL of Master Mix, 0.8 μL of forward primer, 0.8 μL of reverse primer, 2 μL of diluted cDNA, and 6.4 μL of nuclease-free H_2_O. The PCR protocol included an initial denaturation at 95 °C for 30 s, followed by 40 cycles of 95 °C for 10 s and 60 °C for 30 s. A melting curve analysis was conducted to verify amplification specificity. All samples were run in triplicate. The primer sequences are provided in Table 1. Gene expression levels were normalized to β-actin and analyzed via the 2^−ΔΔCT^ method.

**TABLE 1:** Primer sequences used for RT-qPCR.

Gene name	Forward	Reverse
MET	AGC​AAT​GGG​GAG​TGT​AAA​GAG​G	CCC​AGT​CTT​GTA​CTC​AGC​AAC
GAPDH	GTC​AAG​GCT​GAG​AAC​GGG​AA	AAA​TGA​GCC​CCA​GCC​TTC​TC

### 2.11 Western blot

Total protein was extracted from A549 cells, H1299 cells, and tissue samples using lysis buffer (PC101, Epizyme, China) supplemented with protease and phosphatase inhibitors (GRF101/GRF102, Epizyme, China). Protein concentration was determined by NanoDrop, and 30 μg of protein per sample was denatured with loading buffer (LT101S, Epizyme, China) at 100 °C for 5 min. Proteins were separated by SDS-PAGE on 7.5% gels (PG211, Epizyme, China) alongside protein markers (26,616, Thermo Fisher, United States; PL00003, Proteintech, China) at 80–120 V and subsequently transferred to PVDF membranes (IPVH00010, Millipore, United States; 0.45 μm) using a wet transfer system at 300 mA for 40–90 min at 4 °C. Membranes were blocked with 5% skim milk (PS112, Epizyme, China) in TBST for 1 h at room temperature, followed by incubation with primary antibodies overnight at 4 °C.

The primary antibodies used were as follows: AKT (60203-2-Ig, Proteintech, China), p-AKT (4060T, CST, United States), PI3K (60225-1-I, Proteintech, China), p-PI3K (AP0854, ABclonal, China), mTOR (T55306, Abmart, China), p-mTOR (T56571, Abmart, China), MET (25869-1-AP, Proteintech, China), p-MET (30737-1-AP, Proteintech, China), β-actin (66009-1-Ig, Proteintech, China).

After washing, membranes were incubated with HRP-conjugated secondary antibodies (SA00001-1/SA00001-2, Proteintech, China). Protein bands were visualized using an ECL substrate (PK10003, Proteintech, China), and β-actin was used as the loading control for normalization with Image Studio software.

### 2.12 *In vitro* HGF rescue assay

To verify the dependence of 18β-GA-mediated AKT inhibition on MET, HGF rescue experiments were performed. LUAD cells were seeded in culture dishes, grown to 70%–80% confluence, and then serum-starved for 24 h to quiesce basal kinase activity. The cells were divided into four groups. (1) Control group: cells were incubated with serum-free medium containing an equivalent volume of vehicle (0.1% DMSO) for 24 h (2) 18β-GA group: cells were pretreated with 18β-GA at the high concentration determined in Section 2.2 in serum-free medium for 24 h (3) HGF group: cells were incubated with serum-free medium containing vehicle (0.1% DMSO) for 24 h; 15 min before protein extraction, recombinant human HGF (rp175908; Aladdin, China) was added to a final concentration of 50 ng/mL, and the cells were stimulated for 15 min at 37 °C (Yano et al., 2008; Nishimura et al., 2016; Baltschukat et al., 2019). (4) Combination group: cells were pretreated with 18β-GA as in the 18β-GA group for 24 h, and then stimulated with HGF (50 ng/mL, 15 min) in the same manner. After the respective treatments, cells were placed on ice, washed with cold PBS, and lysed for immunoblotting.

### 2.13 *In vivo* SC79 rescue assay

To investigate whether the antitumor effect of 18β-GA was mediated through inhibition of the AKT pathway, a rescue experiment was conducted using SC79 (T2274, TargetMol, United States), a cell-permeable AKT activator (Wen et al., 2020). To Sixteen tumor-bearing mice were randomly assigned to four treatment groups (n = 4; Figure 9A): Model: saline (0.2 mL, ip, daily); 18β-GA HD: 18β-GA (60 mg/kg, ip, daily); SC79: SC79 (5 mg/kg, ip, daily); Combination: SC79 (5 mg/kg, ip) administered 1 h prior to 18β-GA (60 mg/kg, ip, daily).

After 14 days of treatment, mice were euthanized, and tumor tissues were harvested 24 h after the final dose. The tumor tissues were then homogenized, and total protein was extracted for Western blot analysis. The phosphorylation levels of AKT and MET were assessed, and the ratios of p-AKT/AKT and p-MET/MET were quantified to evaluate pathway activity.

### 2.14 Statistical analysis

The data were analyzed and visualized using GraphPad Prism (v10.2.0) software. All experiments were independently repeated at least three times. The experimental results were expressed as mean ± standard deviation (Mean ± SD). Independent samples t-test was used for comparisons between two groups, and one-way ANOVA was used for comparisons among multiple groups. A *p* value <0.05 was considered statistically significant.

## 3 Results

### 3.1 18β-GA inhibits the proliferation of LUAD cells

**FIGURE 1:**
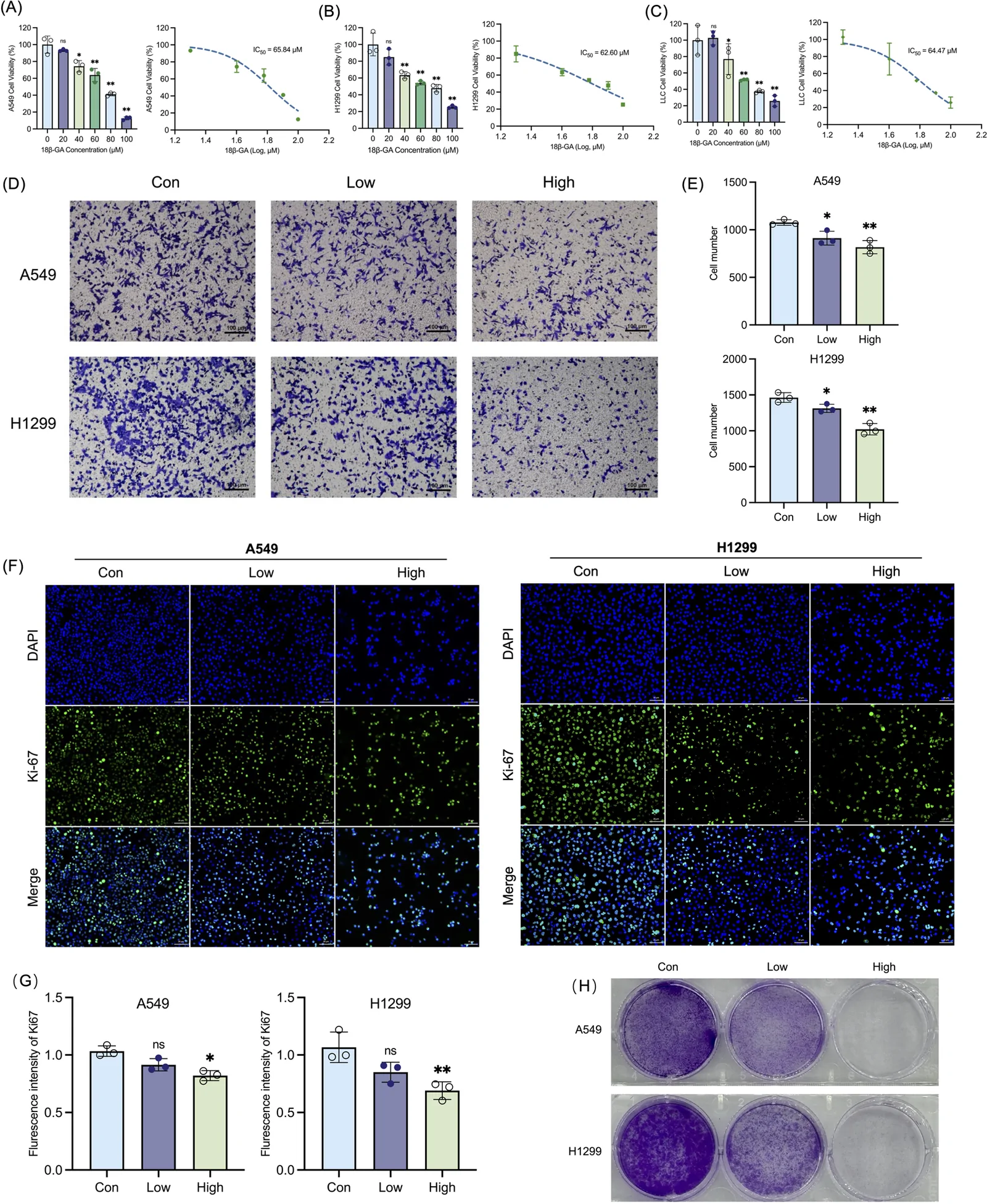
18β-GA inhibits the proliferation of LUAD cells. **(A–C)** Effect of 18β-GA on the viability and proliferation of LUAD cells. Results are expressed as mean ± SD, with n = 3, *p < 0.05, **p < 0.001 vs. 0 μM group. **(D,E)** Transwell migration assay of A549 and H1299 cells following 18β-GA treatment. Scale bar: 100 μm. **(F,G)** Ki67 immunofluorescence staining of A549 and H1299 cells post 18β-GA treatment. Scale bar: 20 μm. Results are expressed as mean ± SD, with n = 3, *p < 0.05, **p < 0.01 vs. control group. **(H)** Colony formation assay of A549 and H1299 cells treated with 18β-GA.

18β-GA exhibited dose-dependent cytotoxicity in A549, H1299, and LLC cells, with IC_50_ values of 65.84, 62.80, and 64.47 μM, respectively (Figures 1A–C). Based on these results, high (65 μM) and low (32.5 μM) doses were selected for A549 and H1299 cells. 18β-GA significantly inhibited cell migration (Transwell; Figures 1D,E), reduced Ki67 expression (immunofluorescence; Figures 1F,G), and decreased clonogenic survival (colony formation; Figure 1H), demonstrating potent anti-migratory and anti-proliferative effects.

### 3.2 18β-GA inhibited LLC allograft tumor growth *in vivo* with a favorable safety profile

**FIGURE 2:**
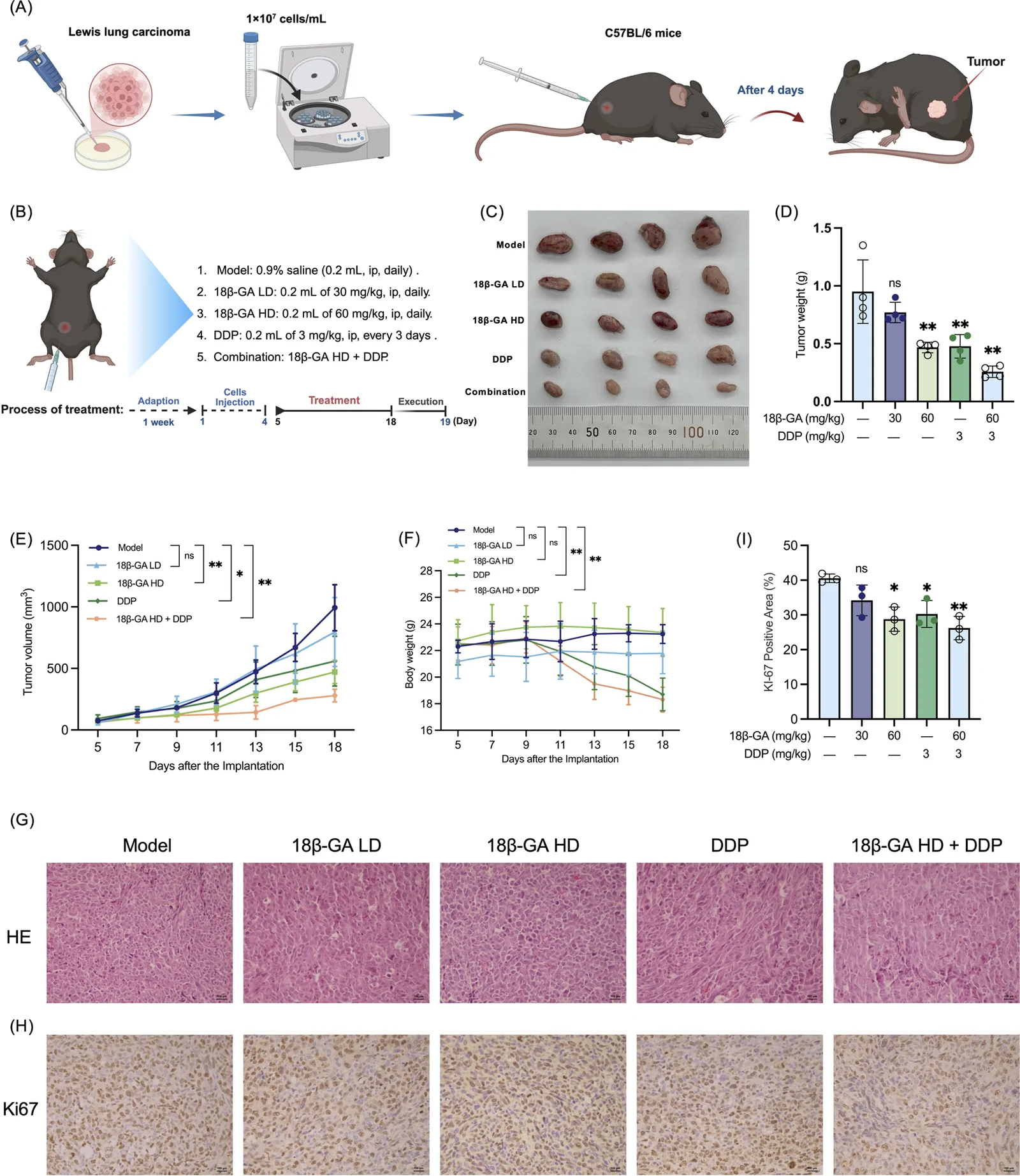
18β-GA inhibits murine LLC allograft tumor growth and *in vivo* safety profile evaluation. **(A)** Schematic of the LLC allograft model establishment. **(B)** Treatment schedule for the LLC allograft model. **(C)** Representative photographs of resected tumors from each group. **(D)** Tumor weights in each group after 14 days of treatment. **(E)** Tumor volume curves of mice in each group throughout the treatment period. **(F)** Body weight curves of mice in each group (an indicator of systemic toxicity). **(G)** H&E staining of tumor sections from each group. Scale bar: 100 μm. **(H,I)** Ki67 IHC staining **(H)** and its quantitative analysis **(I)** in tumor sections. Scale bar: 100 μm. Data are presented as mean ± SD. For D, E, F, **(I)** n = 4 mice per group. For I, n = 3 fields were analyzed per mouse. *p < 0.05, **p < 0.01, ***p < 0.001 vs. model group; ns, not significant.

Following the 2-week treatment, mice were euthanized and tumors collected (Figure 2C). 18β-GA dose-dependently reduced final tumor weight, most notably in the high-dose (HD) and combination (18β-GA HD + DDP) groups (Figure 2D). Tumor volume measurements throughout the study confirmed significant growth inhibition in the 18β-GA HD, DDP, and combination groups versus the model group (*p* < 0.05; Figure 2E). Notably, 18β-GA’s antitumor efficacy was accompanied by minimal systemic toxicity. Body weight remained stable in 18β-GA–treated groups (within ±5% of baseline), similar to the model group. In contrast, DDP monotherapy caused marked weight loss from day 9 (Figure 2F), highlighting 18β-GA’s superior safety profile.

Histopathology further corroborated 18β-GA’s efficacy. H&E staining showed extensive tumor damage, featuring nuclear pyknosis and tissue architecture loss (Figure 2G). IHC analysis revealed a marked decrease in Ki-67-positive cells (Figures 2H,I), confirming potent suppression of proliferation. Collectively, 18β-GA exerts potent and well-tolerated antitumor activity against LLC allografts *in vivo*.

### 3.3 Prediction of key pathways and hub genes for 18β-GA-mediated LUAD suppression by network pharmacology

**FIGURE 3:**
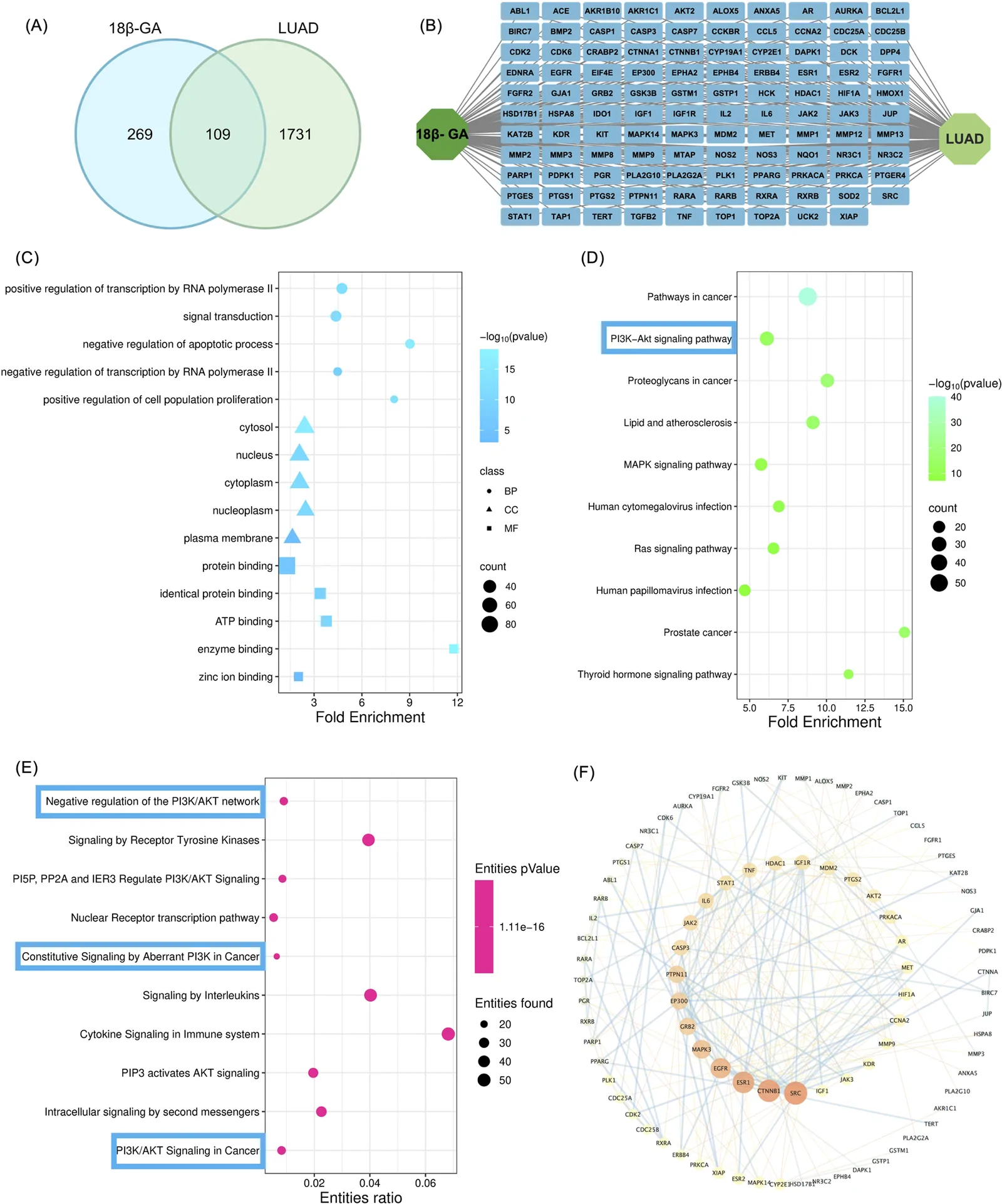
Network pharmacology analysis of 18β-GA and LUAD. **(A)** Venn diagram depicting 18β-GA and LUAD. **(B)** Network diagram of 18β-GA, overlapping targets, and LUAD. **(C)** GO pathway enrichment analysis. **(D)** KEGG pathway enrichment analysis. **(E)** The enrichment analysis results of the REACTOME database. **(F)** Optimized PPI network highlighting crucial targets for 18β-GA and LUAD.

A comprehensive screening approach identified 378 potential binding targets of 18β-GA from pharmacological databases (TCMSP, ETCM 2.0, SwissTargetPrediction, PharmMapper) and 1,840 LUAD-related genes from disease databases (GeneCards, OMIM, DisGeNET). Their intersection revealed 109 overlapping genes (Figure 3A), representing candidate therapeutic targets of 18β-GA in LUAD. A drug-target-disease network constructed in Cytoscape (v3.10.1, Figure 3B) illustrated the multi-target mechanism of 18β-GA and highlighted central hub genes potentially critical to its inhibitory effects.

GO enrichment analysis of these 109 genes identified 1,576 biological processes (BP), 74 cellular components (CC), and 136 molecular function (MF) terms. The top five terms per category are shown in Figure 3C. BP results were primarily associated with transcriptional regulation by RNA polymerase II. CC terms indicated alterations in subcellular localization, including the plasma membrane, cytoplasm, and nucleoplasm. MF analysis implied suppression of proliferation pathways and reversal of apoptosis inhibition.

KEGG analysis revealed 185 enriched pathways, with the top 10 shown in Figure 3D. The PI3K/AKT signaling pathway was identified as a key regulatory axis. REACTOME analysis confirmed this finding, showing significant enrichment in “Negative Regulation of the PI3K/AKT Network” and “PI3K/AKT Signaling in Cancer” (Figure 3E), suggesting that 18β-GA likely suppresses LUAD proliferation via the PI3K/AKT pathway.

A PPI network of the 109 overlapping genes was constructed with STRING (109 nodes, 238 edges; Supplementary Figure S1) and analyzed in Cytoscape. Node sizes were proportional to degree centrality (Figure 3F). Hub genes identified by CytoHubba (Supplementary Table S1) were integrated across algorithms. After duplicate removal, 28 core hub genes were selected for further validation (Supplementary Table S2).

### 3.4 Analysis of TCGA-LUAD transcriptome data and hub genes validation

**FIGURE 4:**
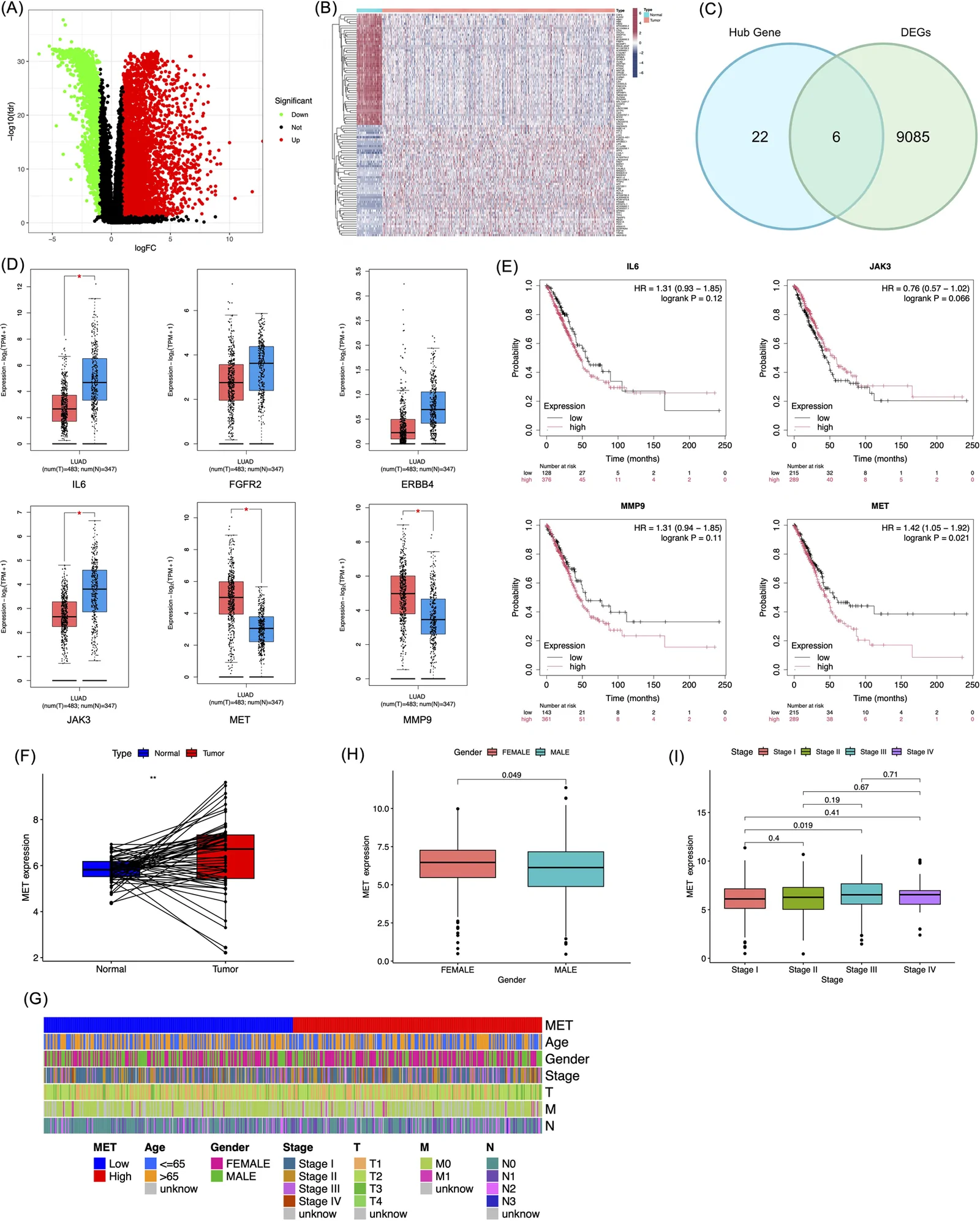
Screening and analysis of TCGA-LUAD transcriptome data. **(A)** The volcano plot illustrates the DEGs between the tumor and normal groups, with green indicating downregulated genes and red indicating upregulated genes. **(B)** Heatmap of the expression levels of the DEGs. **(C)** Venn diagram depicting hub genes and DEGs. **(D)** mRNA expression levels of IL6, FGFR2, ERBB4, JAK3, MET, and MMP9 in normal and tumor tissues from the TCGA-LUAD cohort. *p < 0.05 vs. Normal group. **(E)** Survival analysis of IL6, JAK3, MMP9, and MET. **(F)** Paired differential analysis of MET. **p < 0.01 vs. Normal group. **(G)** Clinical Correlation analysis of MET. **(H)** Analysis of MET expression by gender. **(I)** MET expression levels across clinical stages of LUAD.

Analysis of TCGA-LUAD data identified 9,088 differentially expressed genes (DEGs) (|log_2_FC| > 1, adjusted *p* < 0.05), comprising 6,504 upregulated and 2,584 downregulated genes (Figures 4A,B). Intersection of these DEGs with the 28 hub genes revealed six overlapping candidates: IL6, FGFR2, ERBB4, JAK3, MET, and MMP9 (Figure 4C; Supplementary Table S3). These genes, at the intersection of network pharmacology predictions and LUAD transcriptomic alterations, were selected for further investigation of 18β-GA’s mechanism.

Validation using GEPIA2 and GTEx databases confirmed significant differential expression of IL6, JAK3, MMP9, and MET in LUAD (Figure 4D). Subsequent Kaplan–Meier survival analysis showed that only high MET expression significantly correlated with poor prognosis (log-rank *p* < 0.05; Figure 4E), identifying MET as a key candidate for further study.

Analysis of paired tumor and normal tissues from TCGA-LUAD confirmed MET overexpression in tumors (Figure 4F). Clinically, the overview is shown in Figure 4G. MET expression showed no association with patient age (Supplementary Figure S2) but was significantly higher in females (*p* = 0.049; Figure 4H). No correlation was observed with M, N, or T stage (Supplementary Figures S3-S5); however, MET expression was elevated in advanced overall disease stages (Stage III vs. Stage I, *p* < 0.05; Figure 4I).

These results suggest that MET detection in early-stage (Stage I/II) LUAD could serve as a biomarker for identifying high-risk patients who might benefit from intensified monitoring or targeted therapy, potentially improving prognostic stratification and clinical outcomes.

### 3.5 Molecular docking revealed key interactions between 18β-GA and target proteins

**FIGURE 5:**
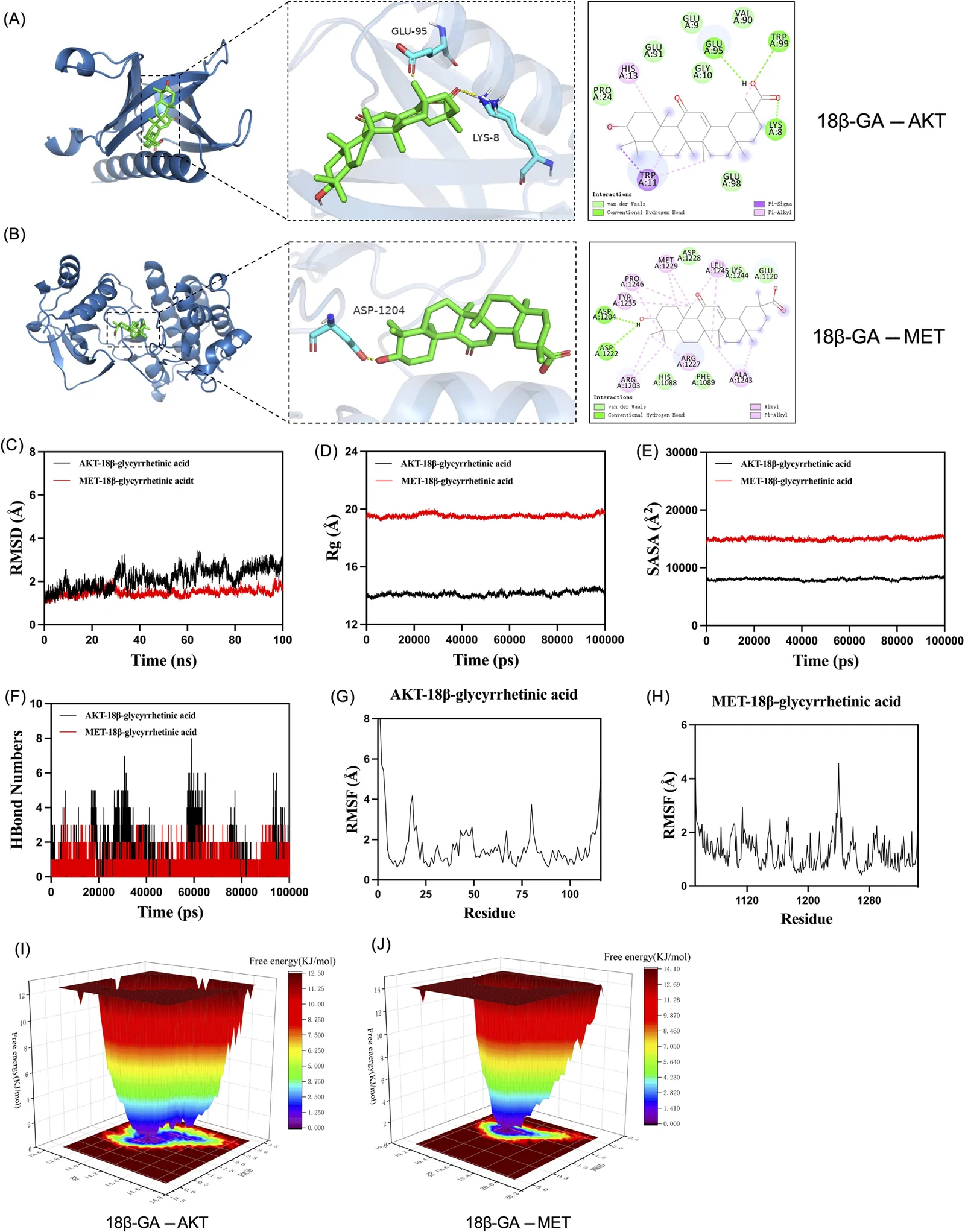
Molecular docking and molecular dynamics simulation. **(A,B)** Molecular docking of 18β-GA to AKT and MET. **(C)** RMSD values of the protein-ligand complex over time. **(D)** Rg values over time. **(E)** SASA values over time. **(F)** HBonds values over time. **(G,H)** RMSF values of the amino acid backbone atoms. **(I,J)** Binding free energy of 18β-GA to AKT and MET.

MET: Glu1120, Lys244, Asp1228, His1088, and Phe1089 were involved in van der Waals interactions; Met1229, Pro1246, Tyr1235, Arg1203, Arg1227, and Ala1243 participated in hydrophobic interactions; Asp1204 and Asp1222 formed hydrogen bonds (Figure 5B; Supplementary Table S4).

AKT: Val90, Gly10, Glu9, Glu91, Pro24, and Glu98 were involved in van der Waals interactions; His13 participated in hydrophobic interactions; Trp99, Glu95, and Lys8 formed hydrogen bonds; Trp11 displayed Pi-Sigma stacking (Figure 5A; Supplementary Table S4).

### 3.6 Molecular dynamics simulations demonstrated system stability

RMSD analysis indicated that the AKT complex stabilized after 85 ns at ∼2.7 Å, while the MET complex reached stability after 90 ns with lower fluctuations, maintaining a value near 1.8 Å (Figure 5C). The Rg exhibited minor variations, indicating consistent compactness during simulation (Figure 5D). SASA changes suggested that ligand binding altered protein surface topology and hydrophobicity (Figure 5E). Hydrogen bond analysis confirmed an average of two bonds in both complexes, with dynamic fluctuations (AKT: 0–8; MET: 0–4), indicating stable binding (Figure 5F). RMSF values (Figures 5G,H) remained low (0.5–2.8 Å), supporting the rigidity of the 18β-GA-MET complex, which occupied a single deep energy well, indicating high stability. In contrast, the 18β-GA-AKT complex sampled multiple low-energy basins, suggesting conformational plasticity during binding (Figures 5I,J).

### 3.7 18β-GA inhibits MET phosphorylation

**FIGURE 6:**
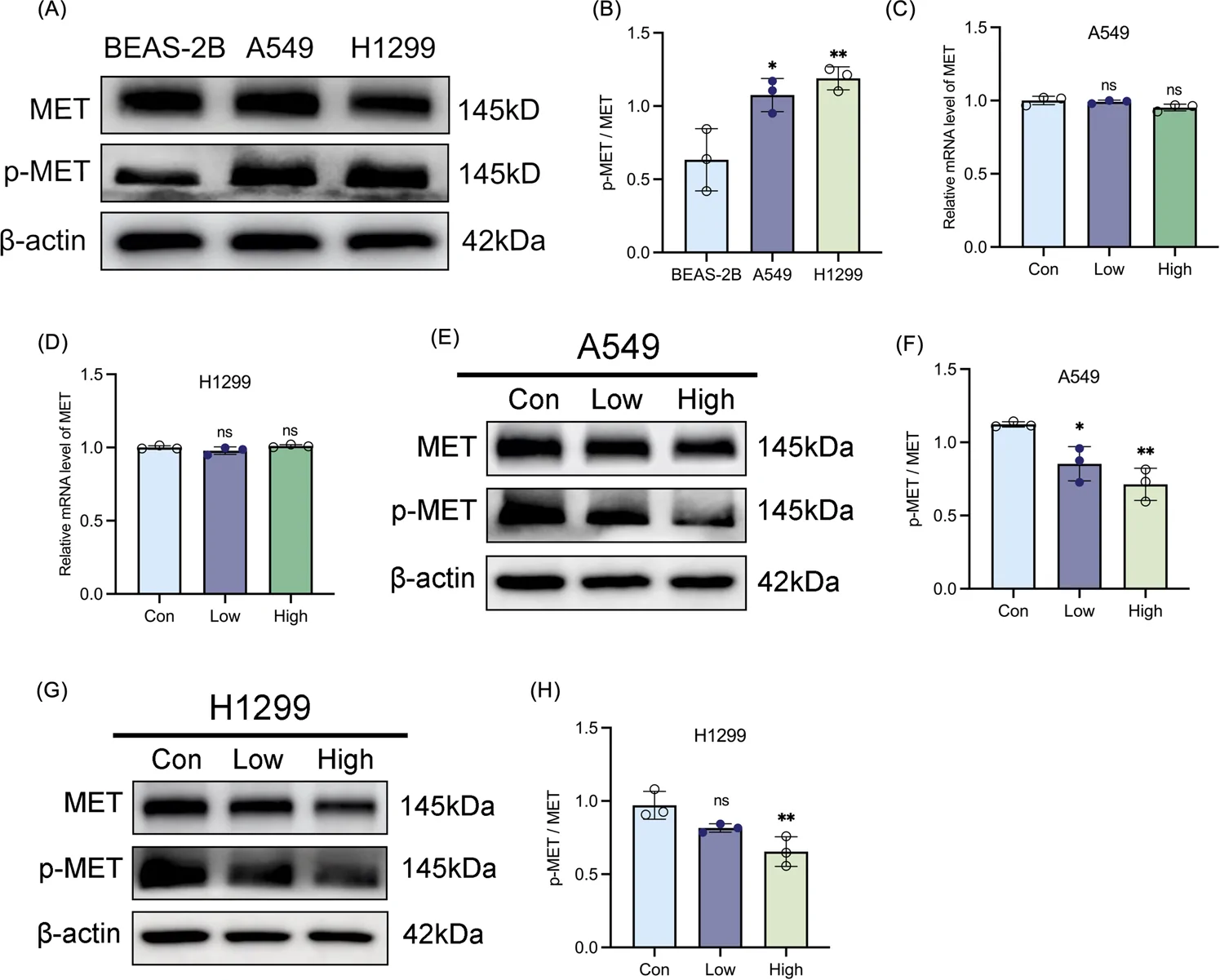
18β-GA inhibits MET phosphorylation in LUAD cells. **(A,B)** Western blot analysis of MET and p-MET protein expression in normal human bronchial epithelial BEAS-2B cells and LUAD cell lines (A549 and H1299). Results are expressed as mean ± SD, with n = 3, *p < 0.05, **p < 0.01 vs. BEAS-2B group. **(C,D)** Relative MET mRNA levels in A549 and H1299 cells were determined by RT-qPCR and normalized to GAPDH. **(E,F)** Western blot analysis of p-MET and MET protein levels in A549 cells. **(G,H)** Western blot analysis of p-MET and MET protein levels in H1299 cells. Results are expressed as mean ± SD, with n = 3, *p < 0.05, **p < 0.01 vs. Control group, ns, not significant. β-Actin was used as a loading control for all Western blot panels.

To validate the potential significance of MET as a target in LUAD as suggested by our transcriptomic analysis, we first assessed its expression and activation status. Interestingly, while bioinformatic predictions indicated an upregulation of MET mRNA in tumor tissues, our experimental data revealed that the total MET protein levels were comparable between the normal bronchial epithelial cell line BEAS-2B and the LUAD cell lines A549 and H1299 (Figures 6A,B). Strikingly, the phosphorylation level of MET (p-MET) was significantly elevated in both cancer cell lines compared to the BEAS-2B cells (Figures 6A,B). We next investigated whether the anti-tumor effect of 18β-GA was mediated through modulation of MET signaling. Treatment with 18β-GA did not alter the mRNA level of MET in either A549 or H1299 cells (Figures 6C,D), effectively ruling out transcriptional suppression as its mechanism of action. However, immunoblotting analysis demonstrated a potent and significant reduction in p-MET levels upon 18β-GA treatment in both cell lines (Figures 6E–H). These results suggest that 18β-GA may directly inhibit the kinase activity of MET or target its upstream regulators to suppress phosphorylation.

### 3.8 18β-GA suppresses LUAD proliferation through inhibition of the PI3K/AKT signaling pathway

**FIGURE 7:**
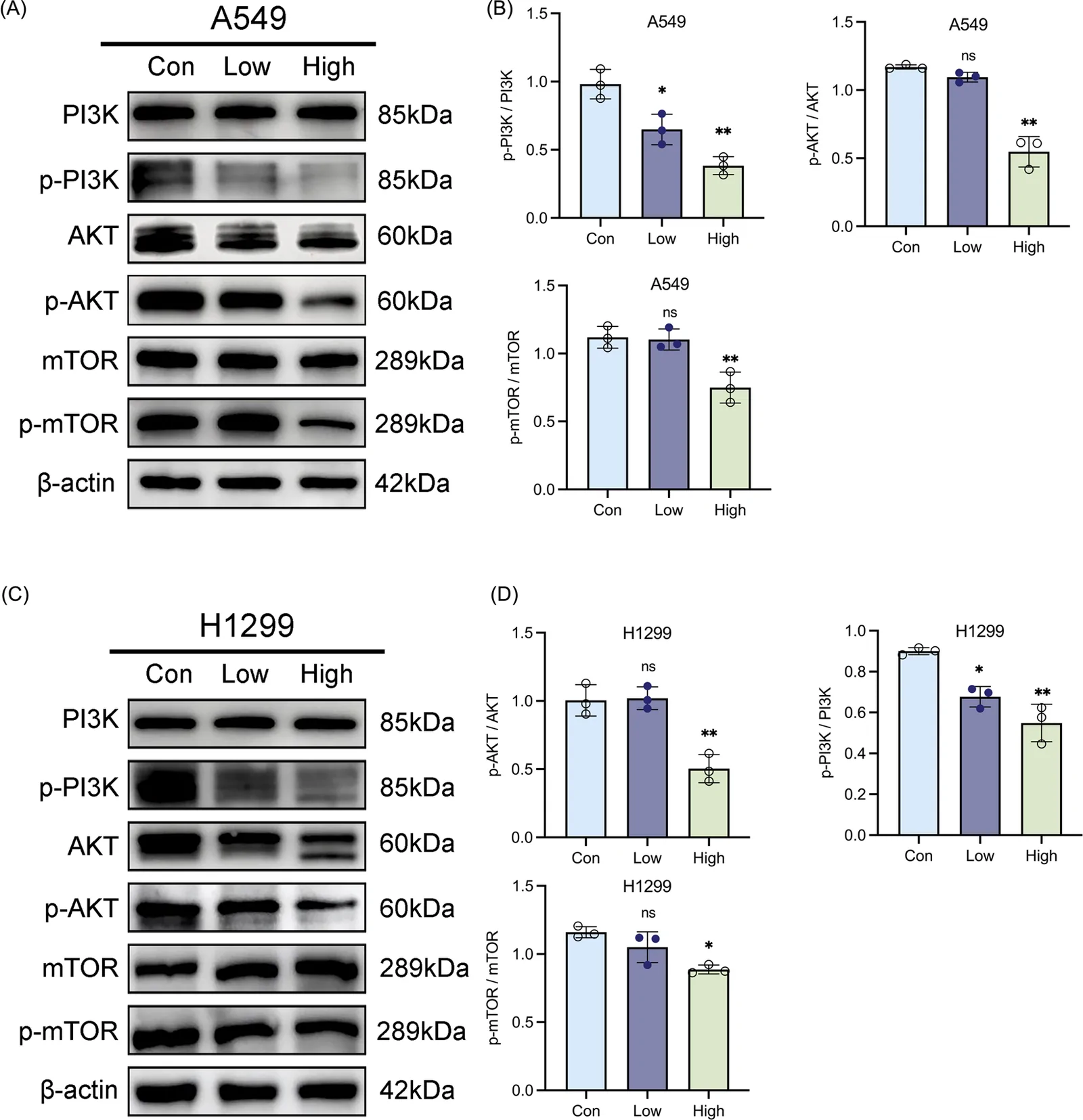
18β-GA inhibits the proliferation of LUAD cells by suppressing the PI3K/AKT signaling pathway. **(A,B)** Western blot analysis of PI3K, p-PI3K, AKT, p-AKT, mTOR and p-mTOR protein levels in A549 cells. **(C,D)** Western blot analysis of PI3K, p-PI3K, AKT, p-AKT, mTOR and p-mTOR protein levels in H1299 cells. β-Actin was used as a loading control for all Western blot panels. All results are expressed as mean ± SD, with n = 3, *p < 0.05, **p < 0.01 vs. Control group, ns, not significant.

To experimentally validate the key pathway identified by our network pharmacology analysis, we examined the effect of 18β-GA on the PI3K/AKT signaling pathway. Consistent with the predictions from our network pharmacology analysis, Western blot analysis demonstrated that 18β-GA treatment potently suppressed the phosphorylation of core pathway components. Specifically, the levels of phosphorylated PI3K (p-PI3K), AKT (p-AKT), and its key downstream target mTOR (p-mTOR) were significantly diminished in both A549 and H1299 cell lines (Figures 7A–D). These data provide strong experimental evidence that inhibition of the PI3K/AKT/mTOR cascade is a central mechanism through which 18β-GA exerts its anti-tumor effects.

### 3.9 Exogenous MET activation reverses 18β-GA-induced inhibition of AKT phosphorylation

**FIGURE 8:**
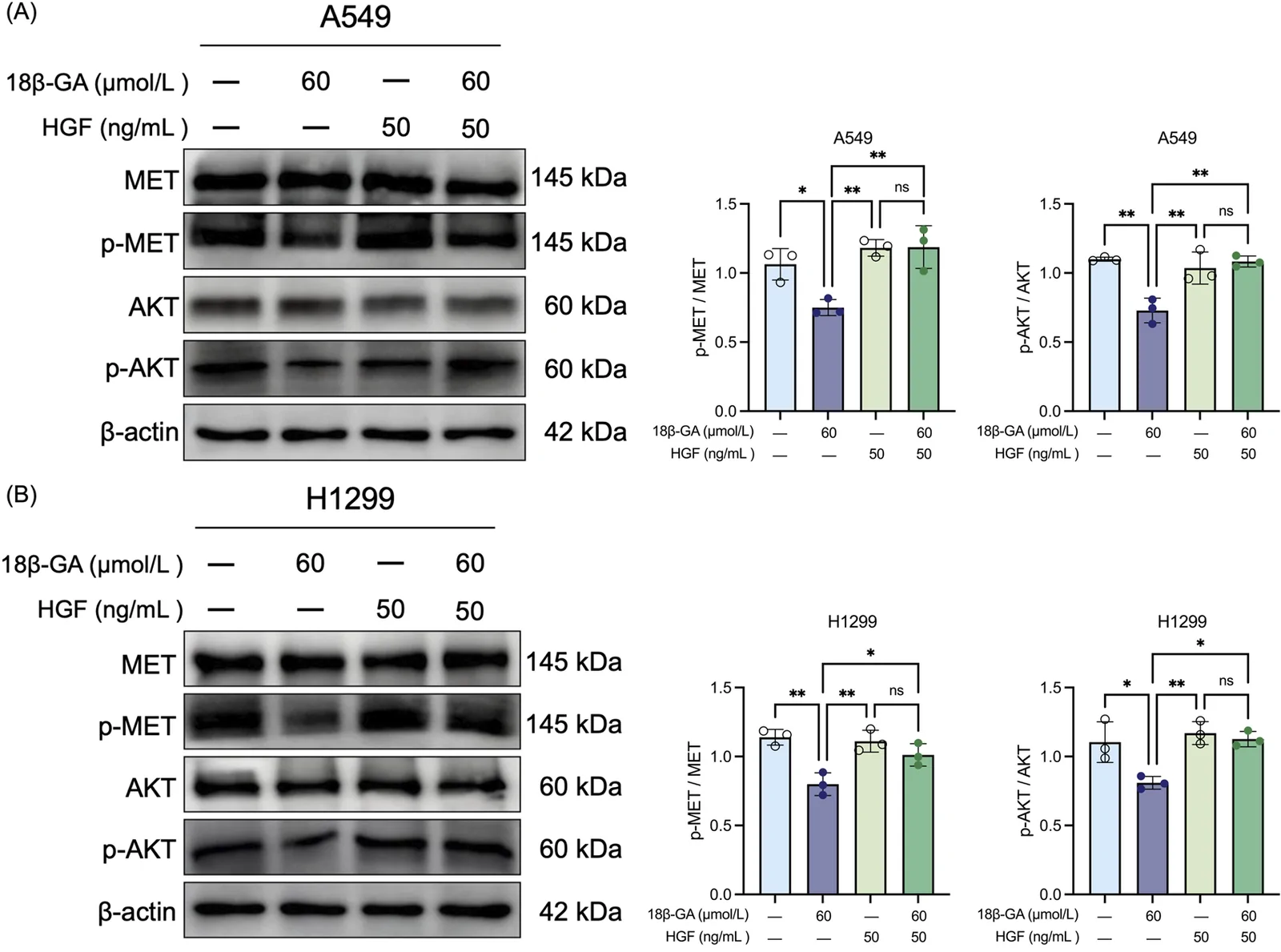
Exogenous MET activation reverses 18β-GA-induced inhibition of AKT phosphorylation. **(A)** Western blot analysis of MET, p-MET, AKT and p-AKT protein levels in A549 cells. **(B)** Western blot analysis of MET, p-MET, AKT and p-AKT protein levels in H1299 cells. β-Actin was used as a loading control for all Western blot panels. All results are expressed as mean ± SD, with n = 3, *p < 0.05, **p < 0.01 vs. Control group, ns, not significant. Created with BioRender.com.

To further elucidate whether the inhibitory effect of 18β-GA on AKT is directly dependent on the inactivation of upstream MET, we performed an *in vitro* functional rescue assay using the MET-specific ligand HGF. Western blot analysis revealed that, compared with the control group, single-agent treatment with 18β-GA significantly decreased the protein levels of p-MET and downstream p-AKT. However, in the combined treatment group, the exogenous addition of HGF not only successfully reactivated MET but also significantly counteracted the 18β-GA-induced inhibition of AKT phosphorylation (*p* < 0.05, Figures 8A,B). These results clearly demonstrate that exogenous MET activation can effectively reverse the 18β-GA-induced inactivation of AKT, thereby directly confirming that 18β-GA blocks downstream AKT signal transduction by inhibiting MET.

### 3.10 SC79-mediated reactivation of AKT rescues the anti-tumor effect of 18β-GA in vivo

**FIGURE 9:**
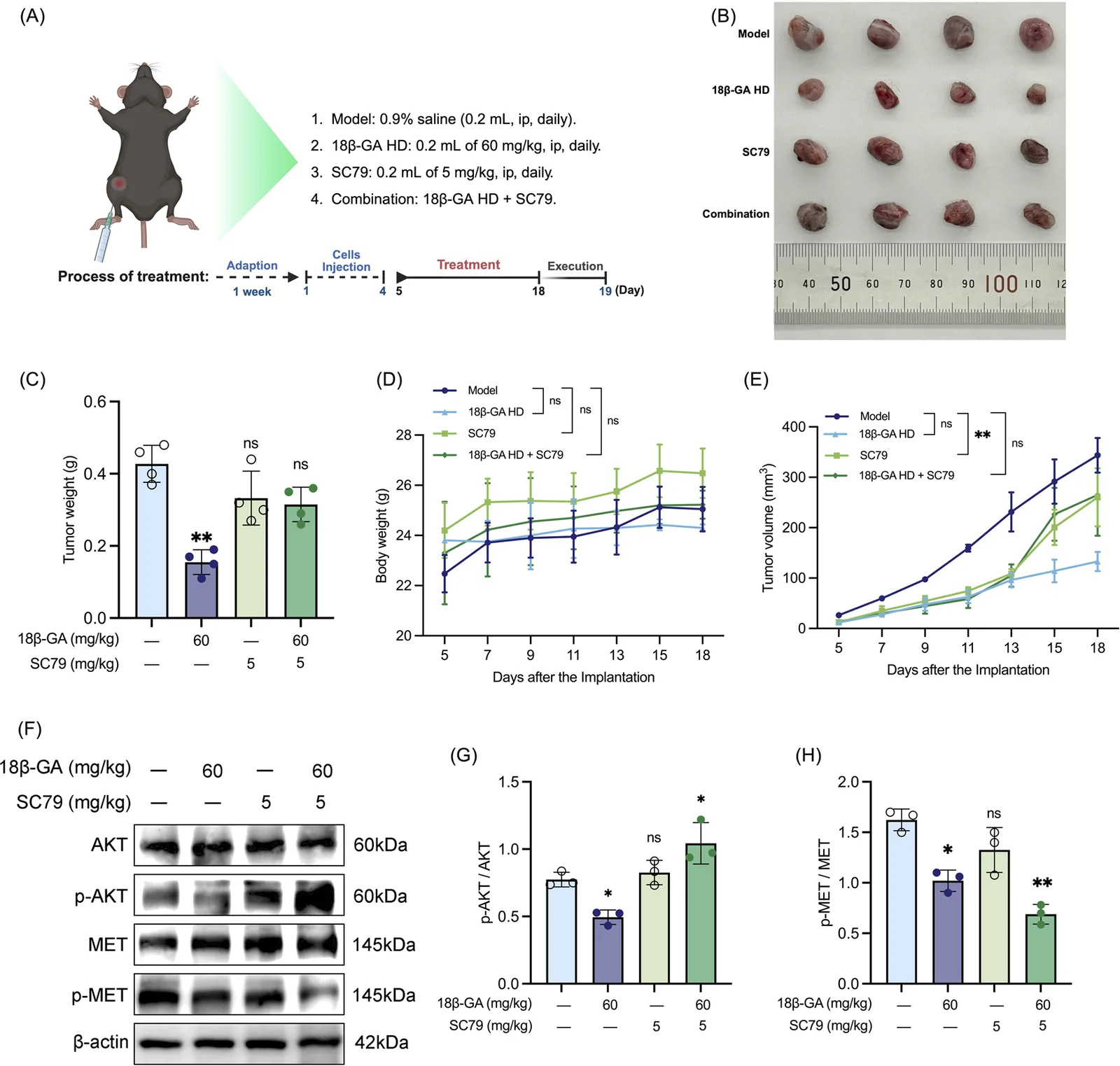
SC79-mediated reactivation of AKT largely reverses the anti-tumor effect of 18β-GA. **(A)** Schematic representation of the treatment process in the Lewis mouse lung cancer model. **(B)** Representative photographs of resected tumors from each group. **(C)** Tumor weights in each group after 14 days of treatment. **(D)** Tumor volume curves of mice in each group throughout the treatment period. **(E)** Body weight curves of mice in each group. **(F–H)** Western blot analysis of AKT, p-AKT, MET and p-MET protein levels in tumor tissues. β-actin was used as a loading control. All data are presented as the mean ± SD. For **(C,D,E)** n = 4 mice per group. For I: n = 3 fields were analyzed per mouse. *p < 0.05, **p < 0.01, ns, not significant. Created with BioRender.com.

To determine if the antitumor effect of 18β-GA depends on AKT signaling, mice received the AKT agonist SC79. After 2 weeks, all mice were weighed, euthanized, and tumors were excised and photographed (Figure 9B). Tumor weights were significantly smaller in the 18β-GA HD group than in the Model, SC79 monotherapy, and combination (18β-GA HD + SC79) groups (*p* < 0.05), which did not differ from each other (Figure 9C). Body weight was comparable across all groups (Figure 9D).

SC79-mediated AKT reactivation markedly reversed 18β-GA’s antitumor effect, as tumor volumes in the combination group increased to approximately 80% of the model group. Accordingly, p-AKT levels suppressed by 18β-GA were restored to baseline (*p* > 0.05) with SC79 co-treatment (Figure 9E). In contrast, 18β-GA–induced suppression of p-MET remained significant (*p* < 0.05) despite AKT reactivation, indicating that MET inhibition is independent of AKT signaling (Figures 9F–H). These results confirm that the PI3K/AKT pathway is essential for 18β-GA’s antitumor activity, while MET inhibition represents a concurrent, independent mechanism.

## 4 Discussion

**FIGURE 10:**
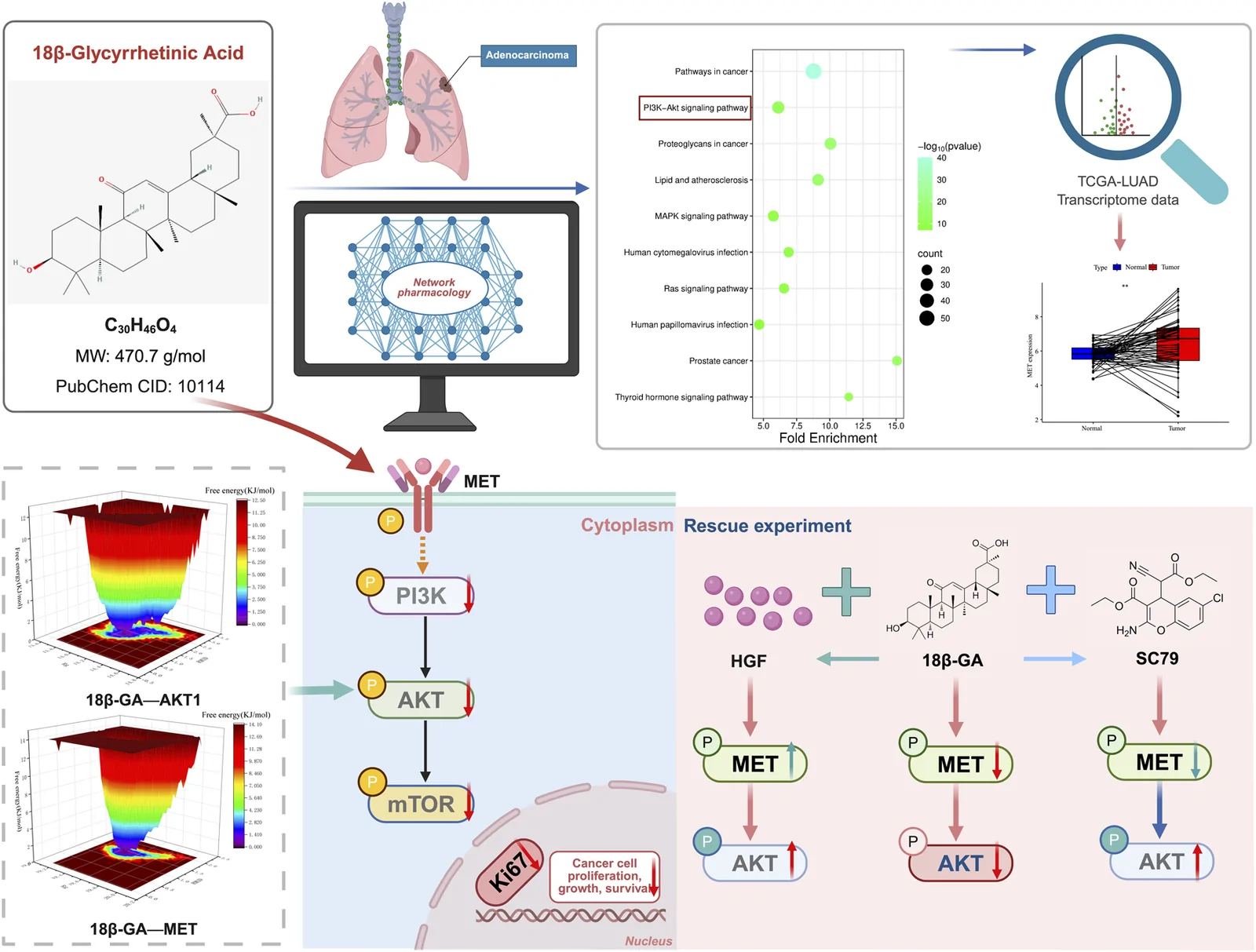
Graphical abstract illustrating the study workflow and proposed mechanism. Created with BioRender.com.

By integrating bioinformatic analyses with *in vitro* and *in vivo* functional assays, this study systematically elucidated the molecular mechanisms by which 18β-GA inhibits LUAD progression via a top-down cascade blockade of the MET/AKT signaling axis (Figure 10). In both cellular and xenograft models, 18β-GA exhibited pronounced anti-proliferative and signaling blockade effects. These findings provide a robust pharmacodynamic basis for developing 18β-GA as a candidate molecule targeting aberrant MET activation, and further highlight the broader potential of natural pentacyclic triterpenoids for precise intervention against tumor-driving kinases.

We initially established its robust efficacy, demonstrating that 18β-GA potently inhibits tumor growth and metastasis both *in vitro* and *in vivo*, while exhibiting a more favorable safety profile than cisplatin. To elucidate the underlying mechanism, we employed an integrated computational approach. Network pharmacology and transcriptomic analysis of LUAD tissues predicted the PI3K/AKT signaling pathway as a central target, with MET emerging as a key upstream regulator. Molecular docking and dynamics simulations further substantiated this finding, revealing that 18β-GA could form stable, high-affinity complexes with both AKT and MET.

Guided by these predictions, we embarked on experimental validation. Intriguingly, while bioinformatics suggested MET mRNA overexpression, our protein-level analysis revealed a more nuanced reality: the oncogenic driver in our LUAD models was not an increase in total MET protein, but rather its marked hyperphosphorylation. We subsequently confirmed that 18β-GA directly targets this activated state, significantly suppressing MET phosphorylation without affecting its mRNA or total protein levels. This cascade of inhibition extended downstream, as 18β-GA treatment also dose-dependently reduced the phosphorylation of PI3K, AKT, and mTOR.

We performed dual rescue experiments using the MET-specific ligand HGF and the AKT agonist SC79 to define the hierarchical regulatory order between MET and AKT. We found that activation of MET by exogenous HGF not only successfully reactivated MET but also significantly reversed the 18β-GA-induced inhibition of AKT phosphorylation and the anti-tumor efficacy of 18β-GA. By contrast, co-treatment with SC79 effectively reversed the tumor-suppressive effects of 18β-GA and restored p-AKT levels, yet failed to abrogate the 18β-GA-induced suppression of p-MET *in vivo*. This unidirectional rescue outcome provides rigorous molecular evidence that MET functions as a critical upstream regulator of AKT.

Notably, aberrant activation of the MET signaling axis is not only a key factor driving the malignant progression of LUAD but also a major mechanism mediating acquired resistance to targeted therapies (Guo et al., 2020). Clinically, although EGFR-TKIs, represented by gefitinib, have significantly improved the prognosis for a subset of patients, the emergence of acquired resistance remains the primary cause of treatment failure (Passaro et al., 2021). Previous studies have shown that following the inhibition of EGFR signaling, MET gene amplification or its aberrant kinase activation can activate bypass signaling (Westover et al., 2018), thereby circumventing TKI-blocked pathways via compensatory activation of critical downstream cascades, such as PI3K/AKT and RAS/RAF/MEK/ERK, and ultimately sustaining tumor cell survival and proliferation (Engelman et al., 2007; Tricker et al., 2015).

This study demonstrated that 18β-GA can block the downstream PI3K/AKT/mTOR signaling cascade by targeting and inhibiting the activation of upstream MET. This intervention modality, which cuts off core survival signals at the source, suggests that 18β-GA holds promise for counteracting resistance signals driven by MET bypass activation, thereby restoring tumor cell sensitivity to EGFR-TKIs. Therefore, validating whether 18β-GA delays or reverses this acquired drug resistance represents a highly translatable follow-up of our present findings. To this end, future studies should establish MET-driven EGFR-TKI-resistant tumor models to systematically assess the synergistic antitumor efficacy of 18β-GA combined with clinically available targeted therapies (e.g., gefitinib, osimertinib) both *in vitro* and *in vivo*. Such follow-up work would not only facilitate the development of innovative combinatorial regimens to overcome bypass resistance triggered by single-agent kinase inhibitors, but also expand the translational prospects of natural small-molecule agents for precision LUAD therapy.

Despite these encouraging findings, several limitations of the present study warrant further investigation. First, although we used the TCGA-LUAD cohort and the GEPIA2 database to confirm the expression and prognostic significance of key targets, the mechanistic scope remains to be expanded. Future work should incorporate transcriptomic profiling of 18β-GA-treated versus untreated LUAD cells, followed by Gene Set Enrichment Analysis (GSEA). This would clarify whether the observed antitumor effects extend beyond the MET/AKT cascade to encompass broader biological processes such as cytokine production, extracellular matrix (ECM) remodeling, TNF signaling, and angiogenesis (Khan and Chakraborty, 2022). Additionally, validating MET upregulation and its association with poor survival in further independent datasets would strengthen the clinical relevance of our findings (Li et al., 2019; Zengin and Önal-Süzek, 2020). Second, with respect to correlation versus causal inference, the HGF rescue experiments provided critical functional support for targeted signaling; however, these results primarily constitute observational evidence derived from biological models. To establish definitive causality while controlling for confounding factors, future studies should integrate transcriptome-wide association studies (TWAS) with Mendelian randomization (MR). By leveraging eQTL and GWAS data, this strategy can systematically evaluate the causal roles of MET and AKT in LUAD prognosis from a genetic perspective. Such analyses should then be complemented by CRISPR-based gene knockout or knock-in functional assays (Zhang et al., 2025). Third, from a statistical modeling perspective, relying on MET and AKT as single-gene biomarkers inherently limits robustness and predictive performance. Future studies could address this by developing multi-gene prognostic signatures, particularly immune-related gene pair models that can effectively mitigate batch effects and platform differences (Xie et al., 2022). This methodology has been shown to yield robust prognostic performance across multiple independent cancer cohorts. Finally, this study did not explore the potential impact of 18β-GA on the tumor immune microenvironment or on the response to immunotherapy. Given the central role of immunotherapy in the clinical management of LUAD, subsequent research should employ single-cell sequencing technologies to characterize how 18β-GA reshapes the immune microenvironment. Moreover, it will be crucial to identify immune subtypes that predict differential responses to 18β-GA combined with immune checkpoint blockade (ICB) and to construct corresponding predictive models for immunotherapeutic efficacy (Zhang et al., 2024; Lai et al., 2025).

In conclusion, this study identifies 18β-GA as a highly promising therapeutic agent against LUAD, exhibiting potent *in vitro* and *in vivo* anti-tumor efficacy coupled with an excellent systemic safety profile. Mechanistically, 18β-GA docks into the core binding pockets of both MET and AKT with stable, low-energy conformations, significantly suppressing their active phosphorylation without altering total protein expression. Crucially, our functional rescue models revealed that 18β-GA halts tumor survival and proliferation by specifically inhibiting the upstream driver MET, thereby driving a “top-down” cascade blockade of the downstream PI3K/AKT/mTOR signaling pathway. Ultimately, the elucidation of this MET/AKT axis-targeted mechanism establishes 18β-GA as a robust and safe signaling interceptor, providing a solid theoretical foundation and a compelling pharmacological rationale for novel LUAD therapeutic strategies, including potential applications in overcoming MET-driven bypass resistance.

## Data Availability

Publicly available datasets were analyzed in this study. This data can be found here: Direct link: https://portal.gdc.cancer.gov/; Repository: The Cancer Genome Atlas (TCGA); Accession number: TCGA-LUAD.

## References

[B1] Aggarwal B. B. Sundaram C. Malani N. Ichikawa H. (2007). Curcumin: the Indian solid gold. Adv. Exp. Med. Biol. 595, 1–75. 10.1007/978-0-387-46401-5_1 17569205

[B2] Baltschukat S. Engstler B. S. Huang A. Hao H.-X. Tam A. Wang H. Q. (2019). Capmatinib (INC280) is active against models of non-small cell lung cancer and other cancer types with defined mechanisms of MET activation. Clin. Cancer Res. Off. J. Am. Assoc. Cancer Res. 25, 3164–3175. 10.1158/1078-0432.CCR-18-2814 30674502

[B3] Cai H. Chen X. Zhang J. Wang J. (2018). 18β-glycyrrhetinic acid inhibits migration and invasion of human gastric cancer cells via the ROS/PKC-α/ERK pathway. J. Nat. Med. 72, 252–259. 10.1007/s11418-017-1145-y 29098529

[B4] Chaitanya Thandra K. Barsouk A. Saginala K. Sukumar Aluru J. Barsouk A. (2021). Epidemiology of lung cancer. Współczesna Onkol. 25, 45–52. 10.5114/wo.2021.103829 33911981 PMC8063897

[B5] Chen L. Gong J. Yong X. Li Y. Wang S. (2024). A review of typical biological activities of glycyrrhetinic acid and its derivatives. RSC Adv. 14, 6557–6597. 10.1039/d3ra08025k 38390501 PMC10882267

[B6] Comoglio P. M. Trusolino L. Boccaccio C. (2018). Known and novel roles of the MET oncogene in cancer: a coherent approach to targeted therapy. Nat. Rev. Cancer 18, 341–358. 10.1038/s41568-018-0002-y 29674709

[B7] De Marco C. Laudanna C. Rinaldo N. Oliveira D. M. Ravo M. Weisz A. (2017). Specific gene expression signatures induced by the multiple oncogenic alterations that occur within the PTEN/PI3K/AKT pathway in lung cancer. PLOS One 12, e0178865. 10.1371/journal.pone.0178865 28662101 PMC5491004

[B8] Efferth T. Fu Y.-jie Zu Y.-gang Schwarz G. Konkimalla V. S. B. Wink M. (2007). Molecular target-guided tumor therapy with natural products derived from traditional Chinese medicine. Curr. Med. Chem. 14, 2024–2032. 10.2174/092986707781368441 17691944

[B9] Engelman J. A. Zejnullahu K. Mitsudomi T. Song Y. Hyland C. Park J. O. (2007). MET amplification leads to gefitinib resistance in lung cancer by activating ERBB3 signaling. Science 316, 1039–1043. 10.1126/science.1141478 17463250

[B10] Fan G. Li D. Liu J. Tao N. Meng C. Cui J. (2024). HNRNPD is a prognostic biomarker in non-small cell lung cancer and affects tumor growth and metastasis via the PI3K-AKT pathway. Biotechnol. Genet. Eng. Rev. 40, 1571–1590. 10.1080/02648725.2023.2196155 36971333

[B11] Guo R. Luo J. Chang J. Rekhtman N. Arcila M. Drilon A. (2020). MET-Dependent solid tumours - molecular diagnosis and targeted therapy. Nat. Rev. Clin. Oncol. 17, 569–587. 10.1038/s41571-020-0377-z 32514147 PMC7478851

[B12] Hasan S. K. Siddiqi A. Nafees S. Ali N. Rashid S. Ali R. (2016). Chemopreventive effect of 18β-glycyrrhetinic acid via modulation of inflammatory markers and induction of apoptosis in human hepatoma cell line (HepG2). Mol. Cell. Biochem. 416, 169–177. 10.1007/s11010-016-2705-2 27116616

[B13] Hsu Y.-C. Hsieh W.-C. Chen S.-H. Li Y.-Z. Liao H.-F. Lin M.-Y. (2023). 18β-glycyrrhetinic acid modulated autophagy is cytotoxic to breast cancer cells. Int. J. Med. Sci. 20, 444–454. 10.7150/ijms.80302 37057216 PMC10087636

[B14] Huang R.-Y. Chu Y.-L. Huang Q.-C. Chen X.-M. Jiang Z.-B. Zhang X. (2014). 18β-Glycyrrhetinic acid suppresses cell proliferation through inhibiting thromboxane synthase in non-small cell lung cancer. PLOS One 9, e93690. 10.1371/journal.pone.0093690 24695790 PMC3973544

[B15] Jiang A.-G. Yu H. Huang J.-A. (2014). Expression and clinical significance of the phosphatidylinositol 3-kinase/protein kinase B signal transduction pathway in non-small cell lung carcinoma. Oncol. Lett. 8, 601–607. 10.3892/ol.2014.2167 25013474 PMC4081182

[B16] Jiang X. Yi S. Shen P. Raj R. Zhang J. Ge H. (2026). Insights on the synergistic effects of glycyrrhetinic acid and isoliquiritigenin on HMGB1-induced inflammation by multi-spectroscopic and molecular docking studies. Spectrochim. Acta, Part A 344, 126726. 10.1016/j.saa.2025.126726 40714661

[B17] Jin L. Zhu Z. Hong L. Qian Z. Wang F. Mao Z. (2023). ROS-Responsive 18β-glycyrrhetic acid-conjugated polymeric nanoparticles mediate neuroprotection in ischemic stroke through HMGB1 inhibition and microglia polarization regulation. Bioact. Mat. 19, 38–49. 10.1016/j.bioactmat.2022.03.040 35415314 PMC8980441

[B18] Jo S. Kim T. Iyer V. G. Im W. (2008). CHARMM-GUI: a web-based graphical user interface for CHARMM. J. Comput. Chem. 29, 1859–1865. 10.1002/jcc.20945 18351591

[B19] Khan S. Chakraborty D. (2022). “Identifying prognostic markers of non-small cell lung carcinoma using bioinformatics,” in Proceedings of International Conference on Industrial Instrumentation and Control. Editors Bhaumik S. Chattopadhyay S. Chattopadhyay T. Bhattacharya S. (Singapore: Springer Nature), 535–545. 10.1007/978-981-16-7011-4_51

[B20] Lai G. Xie B. Zhang C. Zhong X. Deng J. Li K. (2025). Comprehensive analysis of immune subtype characterization on identification of potential cells and drugs to predict response to immune checkpoint inhibitors for hepatocellular carcinoma. Genes. Dis. 12, 101471. 10.1016/j.gendis.2024.101471 40092490 PMC11907441

[B21] Lee M.-C. Kadota K. Buitrago D. Jones D. R. Adusumilli P. S. (2014). Implementing the new IASLC/ATS/ERS classification of lung adenocarcinomas: results from international and Chinese cohorts. J. Thorac. Dis. 6, S568–S580. 10.3978/j.issn.2072-1439.2014.09.13 25349708 PMC4209386

[B22] Lee E. M. Jiménez-Fonseca P. Galán-Moral R. Coca-Membribes S. Fernández-Montes A. Sorribes E. (2023). Toxicities and quality of life during cancer treatment in advanced solid tumors. Curr. Oncol. 30, 9205–9216. 10.3390/curroncol30100665 37887565 PMC10605504

[B23] Li D. Wang Y. Wu H. Lu L. Zhang H. Chang J. (2011). Mitigation of ionizing radiation-induced bone marrow suppression by p38 inhibition and G-CSF administration. J. Radiat. Res. (Tokyo) 52, 712–716. 10.1269/jrr.11007 21971035 PMC3390190

[B24] Li Z. Sang M. Tian Z. Liu Z. Lv J. Zhang F. (2019). Identification of key biomarkers and potential molecular mechanisms in lung cancer by bioinformatics analysis. Oncol. Lett. 18, 4429–4440. 10.3892/ol.2019.10796 31611952 PMC6781723

[B25] Luo Y.-H. Wang C. Xu W.-T. Zhang Y. Zhang T. Xue H. (2021). 18β-Glycyrrhetinic acid has anti-cancer effects via inducing apoptosis and G2/M cell cycle arrest, and inhibiting migration of A549 lung cancer cells. Oncotargets Ther. 14, 5131–5144. 10.2147/OTT.S322852 34712051 PMC8548027

[B26] Mark P. Nilsson L. (2001). Structure and dynamics of the TIP3P, SPC, and SPC/E water models at 298 K. J. Phys. Chem. A 105, 9954–9960. 10.1021/jp003020w

[B27] Mo H. Liu P. (2017). Targeting MET in cancer therapy. Chronic Dis. Transl. Med. 3, 148–153. 10.1016/j.cdtm.2017.06.002 29063069 PMC5643781

[B28] Nagy-Mignotte H. Guillem P. Vesin A. Toffart A. C. Colonna M. Bonneterre V. (2011). Primary lung adenocarcinoma: characteristics by smoking habit and sex. Eur. Respir. J. 38, 1412–1419. 10.1183/09031936.00191710 21828037

[B29] Nishimura Y. Hyuga S. Takiguchi S. Hyuga M. Itoh K. Hanawa T. (2016). Ephedrae herba stimulates hepatocyte growth factor-induced MET endocytosis and downregulation via early/late endocytic pathways in gefitinib-resistant human lung cancer cells. Int. J. Oncol. 48, 1895–1906. 10.3892/ijo.2016.3426 26983447

[B30] Organ S. L. Tsao M.-S. (2011). An overview of the c-MET signaling pathway. Ther. Adv. Med. Oncol. 3, S7–S19. 10.1177/1758834011422556 22128289 PMC3225017

[B31] Passaro A. Jänne P. A. Mok T. Peters S. (2021). Overcoming therapy resistance in EGFR-Mutant lung cancer. Nat. Cancer 2, 377–391. 10.1038/s43018-021-00195-8 35122001

[B32] Sarris E. Saif M. Syrigos K. (2012). The biological role of PI3K pathway in lung cancer. Pharmaceuticals 5, 1236–1264. 10.3390/ph5111236 24281308 PMC3816662

[B33] Scagliotti G. V. Novello S. Von Pawel J. (2013). The emerging role of MET/HGF inhibitors in oncology. Cancer Treat. Rev. 39, 793–801. 10.1016/j.ctrv.2013.02.001 23453860

[B34] Shen K. Cui J. Wei Y. Chen X. Liu G. Gao X. (2018). Effectiveness and safety of PD-1/PD-L1 or CTLA4 inhibitors combined with chemotherapy as a first-line treatment for lung cancer: a meta-analysis. J. Thorac. Dis. 10, 6636–6652. 10.21037/jtd.2018.11.72 30746209 PMC6344734

[B35] Sung H. Ferlay J. Siegel R. L. Laversanne M. Soerjomataram I. Jemal A. (2021). Global cancer statistics 2020: GLOBOCAN estimates of incidence and mortality worldwide for 36 cancers in 185 countries. Ca. Cancer J. Clin. 71, 209–249. 10.3322/caac.21660 33538338

[B36] Tricker E. M. Xu C. Uddin S. Capelletti M. Ercan D. Ogino A. (2015). Combined EGFR/MEK inhibition prevents the emergence of resistance in EGFR-mutant lung cancer. Cancer Discov. 5, 960–971. 10.1158/2159-8290.CD-15-0063 26036643 PMC4824006

[B37] Wang L. He Y. Zhang Y. Zhou H. Yu L. Yang J. (2016). Effects of active components of fuzi and gancao compatibility on bax, Bcl-2, and Caspase-3 in chronic heart failure rats. Evid-based. Compl. Alt. 2016, 7686045. 10.1155/2016/7686045 28053643 PMC5178377

[B38] Wang S. Shen Y. Qiu R. Chen Z. Chen Z. Chen W. (2017). 18 β-glycyrrhetinic acid exhibits potent antitumor effects against colorectal cancer via inhibition of cell proliferation and migration. Int. J. Oncol. 51, 615–624. 10.3892/ijo.2017.4059 28656212

[B39] Wang Z. Chen R. Chen J. Su L. (2024). 18β-glycyrrhetinic acid alleviates radiation-induced skin injury by activating the Nrf2/HO-1 signaling pathway. Biol. Chem. 405, 407–415. 10.1515/hsz-2023-0200 38598859

[B40] Wen L. Wang K. Zhang F. Tan Y. Shang X. Zhu Y. (2020). AKT activation by SC79 to transiently re-open pathological blood brain barrier for improved functionalized nanoparticles therapy of glioblastoma. Biomaterials 237, 119793. 10.1016/j.biomaterials.2020.119793 32044521

[B41] Westover D. Zugazagoitia J. Cho B. C. Lovly C. M. Paz-Ares L. (2018). Mechanisms of acquired resistance to first- and second-generation EGFR tyrosine kinase inhibitors. Ann. Oncol. 29, i10–i19. 10.1093/annonc/mdx703 29462254 PMC6454547

[B42] Xia B. Zhang S. Ma S. (2017). Management of non-small cell lung cancer with EGFR mutation: the role of radiotherapy in the era of tyrosine kinase inhibitor therapy-opportunities and challenges. J. Thorac. Dis. 9, 3385–3393. 10.21037/jtd.2017.09.67 29221323 PMC5708426

[B43] Xie B. Li K. Zhang H. Lai G. Li D. Zhong X. (2022). Identification and validation of an immune-related gene pairs signature for three urologic cancers. Aging (Milano) 14, 1429–1447. 10.18632/aging.203886 35143414 PMC8876921

[B44] Yano S. Wang W. Li Q. Matsumoto K. Sakurama H. Nakamura T. (2008). Hepatocyte growth factor induces gefitinib resistance of lung adenocarcinoma with epidermal growth factor receptor-activating mutations. Cancer Res. 68, 9479–9487. 10.1158/0008-5472.CAN-08-1643 19010923

[B45] Zengin T. Önal-Süzek T. (2020). Analysis of genomic and transcriptomic variations as prognostic signature for lung adenocarcinoma. BMC Bioinf 21, 368. 10.1186/s12859-020-03691-3 32998690 PMC7526001

[B46] Zhang Q. Wang R. Xu L. (2023). Clinical advances in EGFR-TKI combination therapy for EGFR-Mutated NSCLC: a narrative review. Transl. Cancer Res. 12, 3764–3778. 10.21037/tcr-23-956 38192990 PMC10774042

[B47] Zhang Y. Zhang C. He J. Lai G. Li W. Zeng H. (2024). Comprehensive analysis of single cell and bulk RNA sequencing reveals the heterogeneity of melanoma tumor microenvironment and predicts the response of immunotherapy. Inflamm. Res. Off. J. Eur. Histamine Res. Soc. Al 73, 1393–1409. 10.1007/s00011-024-01905-5 38896289

[B48] Zhang C. Shi D. Lai G. Li K. Zhang Y. Li W. (2025). A transcriptome-wide association study integrating multi-omics bioinformatics and mendelian randomization reveals the prognostic value of ADAMDEC1 in Colon cancer. Arch. Toxicol. 99, 645–665. 10.1007/s00204-024-03910-3 39680087

[B49] Zhu J. Chen M. Chen N. Ma A. Zhu C. Zhao R. (2015). Glycyrrhetinic acid induces G1-phase cell cycle arrest in human non-small cell lung cancer cells through endoplasmic reticulum stress pathway. Int. J. Oncol. 46, 981–988. 10.3892/ijo.2015.2819 25573651 PMC4324580

