# Supplementary material for: 18β-glycyrrhetinic acid inhibits lung adenocarcinoma progression by targeting the MET/AKT signaling axis
Source: Front Cell Dev Biol. 2026 Aug 21;14:1752632. doi: 10.3389/fcell.2026.1752632 (PMC13541738; doi:10.3389/fcell.2026.1752632)
Supplement: Supplementary file 1 [file DataSheet1.docx]

Supplementary Material

# 1 Supplementary Figures and Tables

## 1.1 Supplementary Figures

**Supplementary Figure S1** The PPI network of the 109 common targets of 18β-GA and LUAD.

**Supplementary Figure S2** Correlation between MET expression and patient age.

**Supplementary Figure S3** Analysis of MET expression by M stage.

**Supplementary Figure S4** Analysis of MET expression by N stage.

**Supplementary Figure S5** Analysis of MET expression by T stage.

## 1.2 Supplementary Tables

**Supplementary Table S1** Top ten significant genes identified by different analytical methods.

| Method | Gene name | Count |
| --- | --- | --- |
| Betweenness | EP300, PTGS2, CTNNB1, SRC, TNF, IL6, CASP3, MAPK3, ESR1, CDK | 10 |
| Bottleneck | SRC, EP300, IL6, CASP3, CTNNB1, PTGS2, ESR1, MAPK3, CDK2, HDAC1 | 10 |
| Closeness | SRC, CTNNB1, ESR1, MAPK3, EGFR, EP300, TNF, IL6, STAT1, CASP3 | 10 |
| Degree | SRC, CTNNB1, ESR1, EGFR, MAPK3, PTPN11, EP300, GRB2, JAK2, IL6 | 10 |
| DMNC | KDR, ERBB4, MET, AR, MDM2, JAK3, JAK2, CDC25B, CDK2, FGFR2 | 10 |
| Eccentricity | STAT1, SRC, AKT2, MAPK14, MDM2, JAK2, MAPK3, KDR, TNF, MMP9 | 10 |
| EPC | SRC, ESR1, CTNNB1, EGFR, PTPN11, MAPK3, IGF1R, STAT1, GRB2, JAK2 | 10 |
| MCC | SRC, EGFR, PTPN11, JAK2, GRB2, ESR1, IGF1R, CTNNB1, MET, EP300 | 10 |
| MNC | SRC, ESR1, EGFR, MAPK3, CTNNB1, PTPN11, EP300, GRB2, JAK2, IL6 | 10 |
| Radiality | SRC, CTNNB1, EP300, ESR1, MAPK3, STAT1, TNF, EGFR, CASP3, IL6 | 10 |
| Stress | CTNNB1, EP300, SRC, PTGS2, MAPK3, TNFIL6, CASP3, ESR1, EGFR | 10 |

**Supplementary Table S2** Twenty-eight hub genes at the intersection of 18β-GA targets and LUAD-associated genes.

| Gene name | Ensembl gene ID | NCBI gene ID | Protein Name |
| --- | --- | --- | --- |
| PTCS2 | ENSG00000073756 | 5743 | Prostaglandin G/H synthase 2 |
| IGF1R | ENSG00000140443 | 3480 | Insulin-like growth factor 1 receptor |
| FGFR2 | ENSG00000066468 | 2263 | Fibroblast growth factor receptor 2 |
| CTNNB1 | ENSG00000168036 | 1499 | Catenin beta-1 |
| ERBB4 | ENSG00000178568 | 2066 | Receptor tyrosine-protein kinase erbB-4 |
| KDR | ENSG00000128052 | 3791 | Vascular endothelial growth factor receptor 2 |
| MAPK14 | ENSG00000112062 | 1432 | Mitogen-activated protein kinase 14 |
| HDAC1 | ENSG00000116478 | 3065 | Histone deacetylase 1 |
| MET | ENSG00000105976 | 4233 | Hepatocyte growth factor receptor |
| MAPK3 | ENSG00000102882 | 5595 | Mitogen-activated protein kinase 3 |
| JAK3 | ENSG00000105639 | 3718 | Tyrosine-protein kinase JAK3 |
| ESR1 | ENSG00000091831 | 2099 | Estrogen receptor |
| CDC25B | ENSG00000101224 | 994 | M-phase inducer phosphatase 2 |
| IL6 | ENSG00000136244 | 3569 | Interleukin-6 |
| JAK2 | ENSG00000096968 | 3717 | Tyrosine-protein kinase JAK2 |
| AKT2 | ENSG00000105221 | 208 | RAC-beta serine/threonine-protein kinase |
| EGFR | ENSG00000146648 | 1956 | Epidermal growth factor receptor |
| MMP9 | ENSG00000100985 | 4318 | Matrix metalloproteinase-9 |
| CDK2 | ENSG00000123374 | 1017 | Cyclin-dependent kinase 2 |
| MDM2 | ENSG00000135679 | 4193 | E3 ubiquitin-protein ligase Mdm2 |
| PTPN11 | ENSG00000179295 | 5781 | Tyrosine-protein phosphatase non-receptor type 11 |
| AR | ENSG00000169083 | 367 | Androgen receptor |
| STAT1 | ENSG00000115415 | 6772 | Signal transducer and activator of transcription 1 |
| EP300 | ENSG00000100393 | 2033 | Histone acetyltransferase p300 |
| TNF | ENSG00000204490 | 7124 | Tumor necrosis factor |
| CASP3 | ENSG00000164305 | 836 | Caspase-3 |
| GRB2 | ENSG00000177885 | 2885 | Growth factor receptor-bound protein 2 |
| SRC | ENSG00000197122 | 6714 | Proto-oncogene tyrosine-protein kinase Src |

**Supplementary Table S3** The expression of Overlapped genes.

| Group | Gene Symbol | *p*-value |
| --- | --- | --- |
| Upregulated | MMP9 | 2.44E-14 |
|  | JAK3 | 3.42E-16 |
|  | MET | 3.42E-03 |
| Downregulated | IL6 | 1.90E-15 |
|  | FGFR2 | 1.76E-28 |
|  | ERBB4 | 5.12E-26 |

**Supplementary Table S4** Molecular docking result information.

| Target | PDB ID | Ligand | BE (kcal/mol) |
| --- | --- | --- | --- |
| AKT | 1unq | 18β-GA | -7.7 |
| MET | 3zcl |  | -7.6 |
